# Advanced biomaterial-based approaches for medication-related osteonecrosis of the jaw

**DOI:** 10.1515/biol-2025-1346

**Published:** 2026-07-29

**Authors:** Rui Huang, Shiyu Jia, Wei Yin

**Affiliations:** State Key Laboratory of Oral Diseases, National Center for Stomatology, National Clinical Research Center for Oral Diseases, West China Hospital of Stomatology, Sichuan University, Chengdu, Sichuan, 610041, China

**Keywords:** medication-related osteonecrosis of the jaw, biomaterials, nanoparticles, nanotechnology, bioresponsive materials

## Abstract

Medication-related osteonecrosis of the jaw (MRONJ) is a debilitating adverse effect linked to anti-resorptive and anti-angiogenic therapies, with an incidence of 4.34 % (6.22 % in oncology patients and 0.59 % in osteoporosis patients) based on a pooled analysis of 92 observational studies (2015–2020), characterized by persistent bone exposure and poor healing. Conventional treatment modalities often yield suboptimal outcomes, with recurrence and procedural morbidity. In recent years, biomaterial-based strategies have emerged as promising alternatives by targeting MRONJ’s complex pathophysiology, including imbalances in bone remodeling, impaired angiogenesis, immune dysregulation, and infection. This review highlights advances in functional biomaterials for bisphosphonate sequestration, restoration of osteo-angiogenic homeostasis, and immune modulation. These platforms have shown efficacy in promoting tissue regeneration while reducing systemic toxicity. However, clinical translation is limited by biosafety, scalability, and regulatory hurdles, such as instability in the oral environment and mismatched scaffold degradation rates. Future efforts should prioritize multifunctional, intelligent, and patient-specific biomaterials, alongside the establishment of standardized preclinical models and clinical trials. Combinatory strategies have great potential for synergistic treatment and addressing the multiple pathological factors of MRONJ. By integrating materials engineering with mechanistic understanding, this review synthesizes current evidence to highlight the transformative potential of biomaterials for MRONJ prevention and therapy.

## 1 Introduction

Medication-related osteonecrosis of the jaw (MRONJ) is a serious and increasingly recognized complication linked to anti-resorptive and anti-angiogenic therapies. First identified in 2003 in patients receiving bisphosphonates (BPs) [1], MRONJ has become more prevalent due to the widespread use of these agents in managing osteoporosis, Paget’s disease, and bone metastases [2]. According to the American Association of Oral and Maxillofacial Surgeons, MRONJ can be classified into four progressive stages (stages 0 to III) [3]. Clinically, MRONJ manifests as persistent bone exposure accompanied by delayed wound healing, soft tissue inflammation, and, in advanced cases, the development of fistulas, pathological fractures, pain, paraesthesia, and trismus [4], [5], [6]. The condition severely compromises patient quality of life and remains a significant clinical challenge.

**Figure 1:**
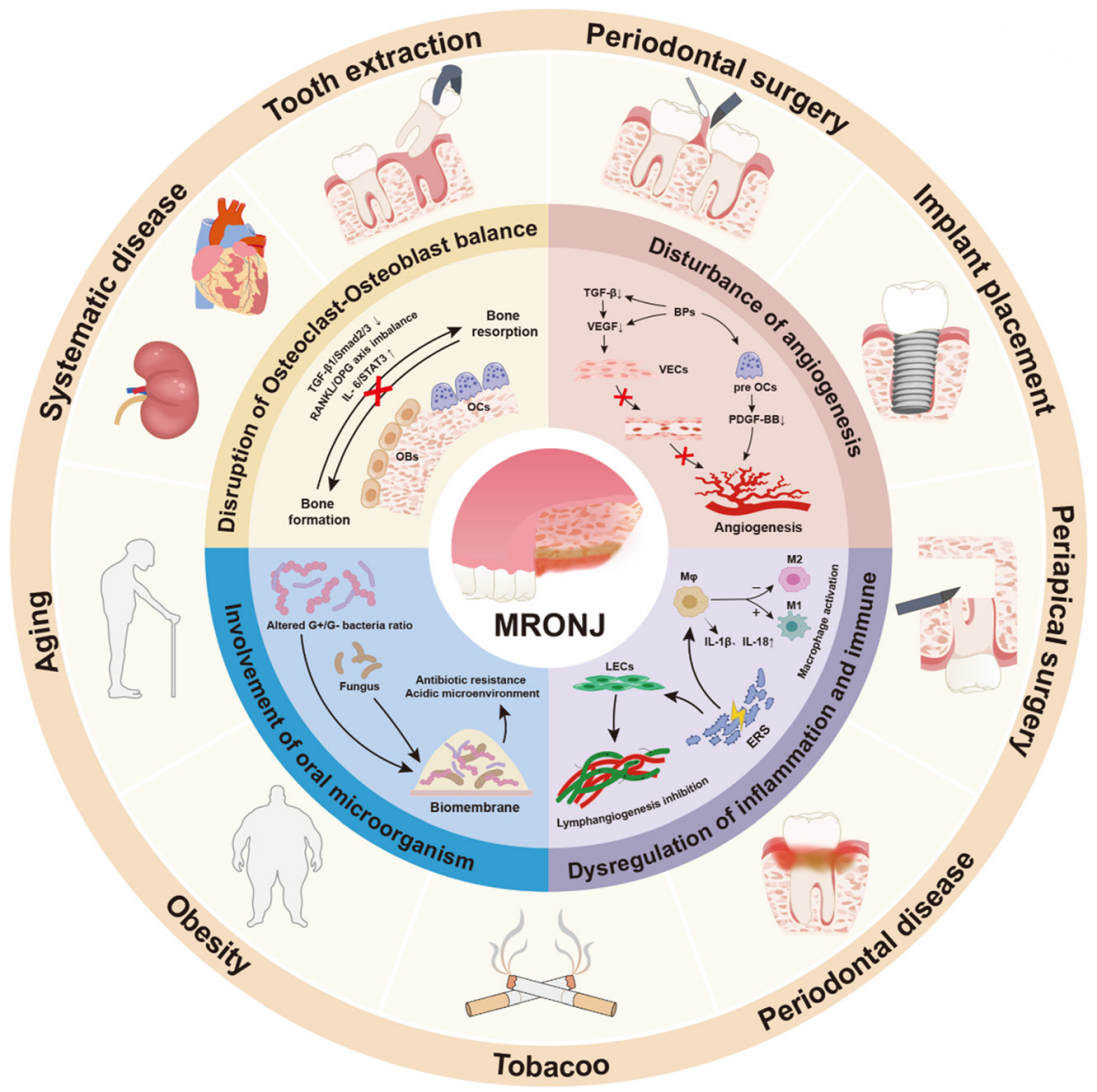
Pathogenic mechanisms and risk factors of MRONJ. The development of MRONJ involves multiple interrelated mechanisms, including impaired bone remodeling, disrupted angiogenesis, immune dysregulation, and microbial colonization. Several risk factors contribute to disease onset, notably invasive dental procedures (e.g., tooth extraction, implant placement) and patient-specific conditions such as systemic comorbidities and medication history. MRONJ, medication-related osteonecrosis of the jaw; TGF-β, transforming growth factor-β; VEGF, vascular endothelial growth factor; BPs, bisphosphonates; PDGF-BB, platelet-derived growth factor-BB; LECs, lymphatic endothelial cells.

Several pharmacologic agents are implicated in MRONJ through distinct but converging mechanisms. BPs and denosumab are the primary antiresorptives associated with this condition. BPs, synthetic analogs of pyrophosphate, become incorporated into the bone matrix and induce osteoclast apoptosis by inhibiting farnesyl pyrophosphate synthase [7]. Denosumab, a monoclonal antibody targeting receptor activator of nuclear factor κB ligand (RANKL), suppresses osteoclastogenesis and bone resorption by blocking RANKL-mediated signaling [8]. Antiangiogenic drugs, including anti-vascular endothelial growth factor (VEGF) monoclonal antibodies (e.g., bevacizumab), decoy receptors (e.g., aflibercept), and tyrosine kinase inhibitors (e.g., sunitinib, cabozantinib, sorafenib), further contribute to MRONJ by impairing vascularization and bone regeneration [9]. Additional contributors include conventional chemotherapeutics, mTOR inhibitors, and immunotherapies, potentially by harming bone homeostasis and mucosal immune responses through immunosuppressive or cytostatic actions, though their mechanisms remain less clearly defined [10], [11], [12], [13]. These drugs collectively impair bone remodeling, increase infection susceptibility, and may exhibit additive effects on MRONJ risk when used in combination [10]. Systemic drug-induced suppression of bone remodeling and immune function may predispose affected sites to impaired healing following local trauma such as tooth extraction, thereby triggering MRONJ onset [6], 14], 15]. Under normal conditions, post-extraction healing progresses through clot stabilization, granulation tissue formation, and bone remodeling over roughly three months [4]. BPs disrupt this process by impairing osteoclast-mediated resorption, thereby delaying wound resolution and increasing the risk of secondary infection and necrosis [4], 16]. Other invasive procedures such as periodontal surgery, dental implants, and periapical surgery are also associated with elevated risk [6]. Among cancer patients with MRONJ, pre-existing inflammatory dental conditions were implicated in 50 % of cases, and therefore, preventive dental care should focus on optimizing oral hygiene (via inflammation control and infection management) to eliminate the necessity for future invasive treatments [10]. Notably, coexisting periodontal disease, dental inflammation, and systemic comorbidities (e.g., diabetes, rheumatoid arthritis, immunosuppression) further exacerbate susceptibility, as do behavioral and demographic factors such as smoking, anemia, advanced age, and obesity (Figure 1) [10], [17], [18], [19].

Current therapeutic approaches include both surgical and non-surgical strategies. Surgical resection of necrotic bone is commonly reserved for advanced MRONJ (Stages II and III) or after failure of conservative therapy, and is associated with significant trauma, morbidity, and variable outcomes [20], [21], [22]. Conservative treatments emphasize infection control via meticulous oral hygiene, antiseptic rinses, and systemic antibiotics [5], 20], 23]. These approaches may stabilize early-stage MRONJ (Stages 0 and I), but often yield limited success due to the spread of antibiotic resistance genes among the oral biofilm inhabitants and potential side effects from prolonged antibiotic use [22]. Adjunctive therapies such as hyperbaric oxygen (HBO) and teriparatide have also been explored. HBO improves oxygen delivery and healing capacity in necrotic tissues, particularly in non-alveolar sites, though its efficacy is highly protocol-dependent and varies by MRONJ stage [24], [25], [26]. Teriparatide, an osteoanabolic agent derived from human parathyroid hormone (1–34), promotes bone remodeling and may serve as a surgical adjuvant; however, evidence of its clinical benefits remains limited. However, Teriparatide is contraindicated in patients with bone metastases and skeletal malignancies, hypercalcemia, urolithiasis, or known hypersensitivity, etc [27], 28]. Overall, no definitive or universally effective treatment for MRONJ has been established [4].

The management of MRONJ represents a significant clinical unmet need, driven by suboptimal patient outcomes, substantial morbidity, and a considerable economic burden. The disease progression, as defined by AAOMS staging, correlates with escalating clinical impact: from asymptomatic bone exposure in early stages (Stages 0 and I) to pain, infection, and pathological fractures in advanced disease (Stages II and III), severely impairing oral function, nutrition, and quality of life [29], 30]. This morbidity translates into a high cost burden, stemming from repeated specialist consultations, long-term antibiotic regimens, complex surgeries, and hospital admissions [29]. There is a pressing priority to develop highly targeted, highly effective, less invasive, more accessible to patients, and more affordable therapeutic strategies.

**Figure 2:**
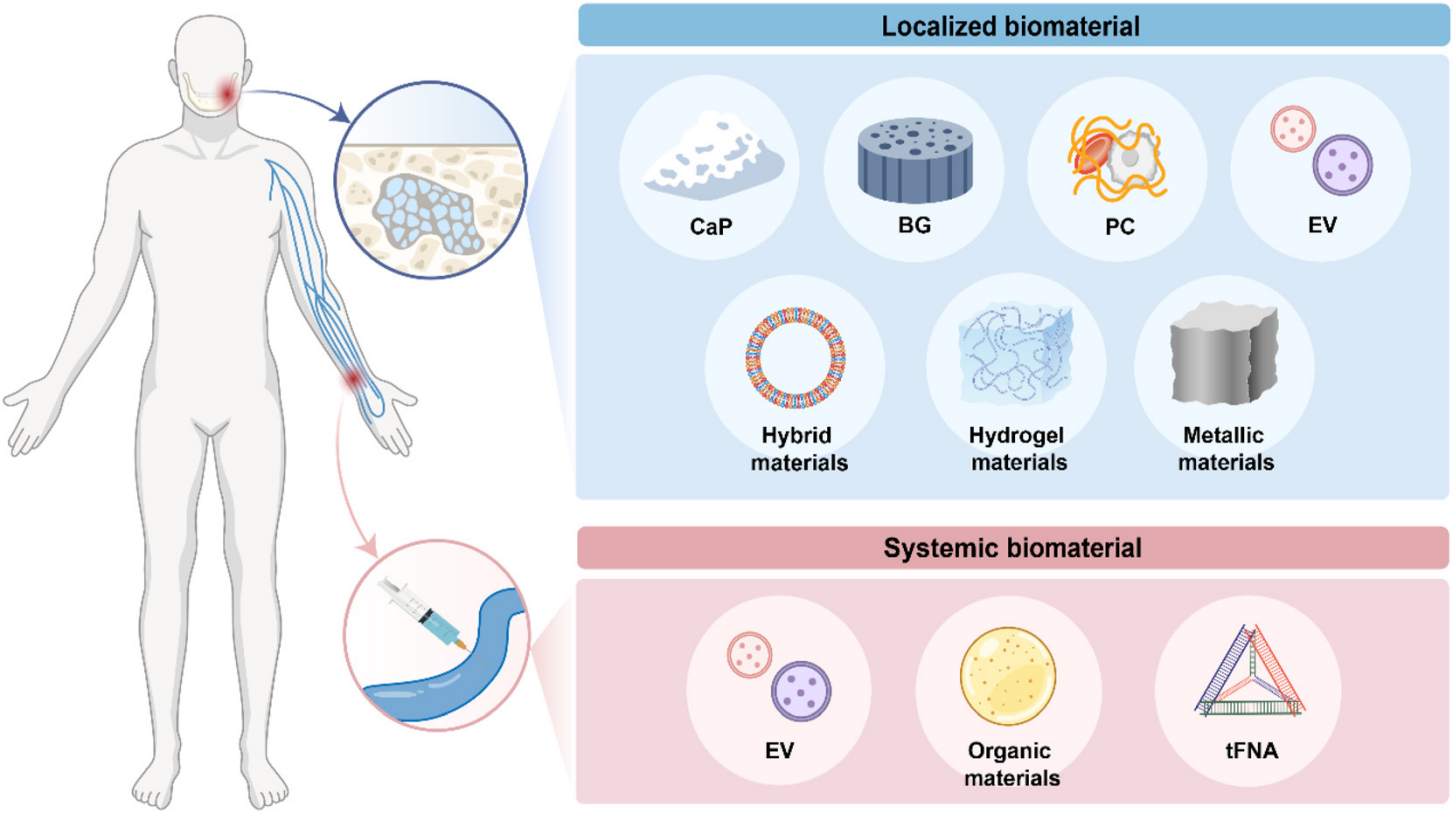
Biomaterial-based strategies for the management of MRONJ. Biomaterials can be applied through local or systemic delivery routes to prevent or treat MRONJ. These approaches aim to enhance site-specific therapeutic effects by promoting bone regeneration, angiogenesis, immune modulation, or drug sequestration depending on the route of administration and biomaterial properties. MRONJ, medication-related osteonecrosis of the jaw; CaP, calcium phosphate; BG, bioactive glass; PC, platelet concentrate; EV, extracellular vesicle; tFNA, tetrahedral framework nucleic acid.

Given the limitations of existing treatments and the multifactorial nature of MRONJ pathogenesis, innovative strategies are urgently needed. In this context, biomaterials offer a promising avenue (Figure 2). Biomaterials provide mechanistic advantages over systemic therapies by enabling localized, multifunctional delivery that directly addresses the therapeutic gap of off-target toxicity and invasive surgical trauma, while promoting tissue integration and controlled release. With their adaptability, bioactivity, and compatibility, advanced biomaterials can be engineered to target specific pathophysiological processes, including drug sequestration, immune modulation, and promotion of osteo-angiogenic coupling. These materials offers a promising alternative that combines spatial precision, controlled release kinetics, and microenvironmental targeting in both preventive and therapeutic approaches, compared to traditional systemic drugs and invasive surgical interventions (Table 1).

**Table 1:** Preclinical studies on biomaterial-based strategies for “MRONJ prevention and treatment” for clarity and scope.

Primary material	Agents	Experimental model	Biological effect	Potential mechanisms	Ref.
β-TCP	/	Rat	Improved epithelial coverage and new bone formation	Absorb free BPs and modulate local autophagy mechanisms	[31]
BCP	/	Rat	Improved healing and tissue maturity	Absorb free BPs and decrease its availability	[32]
β-TCP	BMP-2	Mouse	Increased bone filling ratio, reduced bone necrosis was reduced	Promote bone formation	[33]
β-TCP	E-rhBMP-2	Mouse	Restored osteocyte network and reduced microcracks	Promote bone formation	[34]
BBG	/	Rat	Increased BMD, BV/TV, and Tb·N, decreased necrotic bone area and empty osteocyte lacunae	Promote bone formation and angiogenesis	[35]
F18 bioglass	/	Rat	Recovered bone volume, less gingival ulceration, osteonecrosis, osteolysis, osteomyelitis, bacterial colonies, and abscesses	Exhibit cytoprotection and antibacterial effect	[36]
StarPEG-heparin hydrogel	RGD tripeptide, rhBMP-2	Rat	Increased bone volume and osteoclasts/field, decreased empty lacunae	Promote bone formation	[37]
Gelatin-hyaluronic acid hydrogel	VEGF	Rat	Decreased necrotic area, increased vascular markers vWF and CD105	Modulate angiogenesis and inflammation	[38]
Magnesium	/	Rat	Increased BV/TV and BMD, vessel volume and new bone formation	Promote angiogenesis	[39]
PBM hydrogel	/	Rat	Improved number of type H vessels and Osterix+ osteoblastic precursor cells, increased BV/TV and BMD, reduced abundance of Bacteroidota and recovered population levels of beneficial bacterial groups	Promote bone formation and angiogenesis, modulate inflammation	[40]
CMCS-PMCM-41-NMCM-41 hydrogel	CDM, GGOH	RAW264.7, hMSCs	Sustained antibacterial efficacy against *Streptococcus sanguinis* for 14 days, effective cytoprotection for osteoclastic and osteoblastic progenitors against zoledronic acid toxicity	Exhibit cytoprotection and antibacterial effect	[41]
HepMA hydrogel	PRF	Rat	Reduced open wounds, improved blood vessels and bone formation, decreased inflammation and empty lacunae, increased BV/TV, BMD, Tb·Th and decreased Tb.Sp,	Promote bone formation and angiogenesis	[42]
sEV-AT	/	Rat	Increased BV/TV, Tb·N, Tb·Th, and decreased Tb.Sp, less necrotic bones and empty osteocyte lacuna, increased number of osteoclasts, blood vessels and collagen fibers, promoted tube formation and migration of HUVEC	Promote bone formation and angiogenesis	[43]
MSC-EVs	/	Rat	Improved socket healing with soft tissue, increased bone mass/total tissue mass (%)	Suppress osteoblast, stem cell, and fibroblast aging	[44]
ADSC-exosomes	/	Mouse	Improved bone formation and complete mucosa coverage, increased BV/TV, BMD, and Tb·Th	Promote bone formation and modulate inflammation	[45], 46]
ZDPR	ZA, rapamycin	Mouse	Increased BV/TV, BMD, improved bone formation and lymphatic vessel morphology and function	Modulate inflammation	[47]
tFNA	/	Rat	Increased BV/TV, BMD, more continuous trabeculae	Promote bone formation and exhibit cytoprotection effect	[48]
tFNA-KLT	tFNA-KLT	Rat	Increased BV/TV, better soft tissue healing, improved new bone formation	Promote angiogenesis	[49]

BPs, bisphosphonates; β-TCP, β-tricalcium phosphate; BCP, biphasic calcium phosphate; BMP-2, bone morphogenetic protein-2; E-rhBMP-2, *Escherichia coli*-derived recombinant human BMP-2; BBG, borate bioactive glass; BMD, bone mineral density; BV/TV, bone volume/total volume; Tb·N, trabecular thickness; StarPEG, star-shaped polyn (ethylene glycol); VEGF, vascular endothelial growth factor; PBM hydrogel, poly (ethylene glycol)-bovine serum albumin/arginylglycylaspartic acid-magnesium oxide hydrogel; CMCS-PMCM-41-NMCM-41, carboxymethyl chitosan-plasma-treated mesoporous silica nanoparticles-amine-functionalized mesoporous silica nanoparticles; CDM, clindamycin hydrochloride; GGOH, geranylgeraniol; hMSCs, human mesenchymal stromal cells; HepMA hydrogel, methacrylated gelatin/methacrylated heparin hydrogel; PRF, platelet rich fibrin; Tb.Sp, trabecular separation; AT-SVF, autologous adipose-tissue stromal vascular fraction; sEV-AT, small extracellular vesicles derived from adipose tissue; HUVEC, human umbilical vein endothelial cells; MSC-EVs, mesenchymal stromal cell-derived extracellular vesicles; ADSC, adipose-derived mesenchymal stromal cells; ZDPR, Zoledronic acid acid-1, 2-Distearoyl-sn-glycero-3-phosphoethanolamine-poly(ethylene glycol)-rapamycin nanoparticles; ZA, zoledronic acid acid; tFNA, tetrahedral framework nucleic acids.

This review aims to synthesize the latest advances in biomaterial-based strategies for MRONJ prevention and therapy, with a specific focus on how material design is informed by, and can modulate, key pathogenic mechanisms. The scope encompasses a broad range of biomaterial platforms (e.g., hydrogels, scaffolds, nanoparticles) designed for localized mandibular applications. First, we outline the established and emerging pathophysiological mechanisms of MRONJ, identifying critical therapeutic targets for biomaterial intervention. Second, we categorize and analyze current biomaterial strategies, elucidating the design principles that link material properties (e.g., composition, drug release kinetics, bioactivity) to specific mechanistic targets. Finally, we examine key translational barriers, such as limited material retention, suboptimal drug release, and safety concerns, and propose future directions including bioresponsive systems, patient-specific design, and multifunctional composite strategies. By bridging material science with mechanistic insights, this work aims to guide the development of next-generation therapies for MRONJ.

## 2 Pathogenic mechanisms of MRONJ

Given the multifactorial nature of MRONJ, a clearer understanding of its pathogenesis is essential for guiding rational biomaterial design. The MRONJ remains incompletely understood and is widely regarded as multifactorial. Several interrelated mechanisms have been proposed, including impaired bone remodeling, inhibition of angiogenesis, immune dysregulation, and colonization by oral microbiota (Figure 1). These processes may act synergistically, contributing to the onset and progression of MRONJ. While no single pathway fully accounts for the disease, accumulating evidence supports a composite model that underscores the intricate interplay and feedback mechanisms involving disruptions in skeletal, vascular, immune, and microbial homeostasis, that emphasizes the simultaneous involvement of multiple pathological mechanisms. This imbalance can manifest clinically as delayed socket healing or nonunion, highlighting the need for therapeutic strategies that address these multifactorial pathologies.

### 2.1 Disruption of osteoclast–osteoblast balance

Dysregulation of the osteoclast–osteoblast axis during bone remodeling is widely regarded as a central mechanism in the pathogenesis of MRONJ, leading to exposed necrotic jawbone, sequestra formation and delayed socket healing. Under normal physiological conditions, bone remodeling is a tightly coordinated process in which osteoclast-mediated bone resorption is followed by osteoblast-driven bone formation to restore skeletal integrity [50], 51]. Antiresorptive agents disrupt this balance primarily by inducing osteoclast apoptosis and inhibiting osteoclast differentiation and function [50]. In addition to suppressing bone turnover, this inhibition impairs the clearance of necrotic bone, leading to its accumulation and contributing directly to MRONJ development [52], 53].

At the molecular level, several signaling pathways have been implicated in this imbalance. The transforming growth factor-β (TGF-β) pathway plays a key role in bone matrix synthesis and osteoblast differentiation. BPs have been shown to inhibit TGF-β1 signaling and its downstream mediators Smad2/3 in oral mucosa specimens from 60 MRONJ patients and in animal models, thereby delaying alveolar bone healing [54], 55]. Moreover, BPs is proposed to potentially indirectly suppress osteoclast regulation by downregulating osteoblast-derived RANKL through TGF-β1 inhibition *in vitro*, thereby altering the RANK/RANKL/OPG axis [55], 56]. Animal studies have demonstrated that exogenous application of RANKL can promote osteoclastogenesis and improve extraction socket healing even in the presence of BPs; On the other hand, when osteoclast precursors were cultured with osteoblasts, it seemed that osteoclast activation was attenuated especially when they were stimulated with high concentrations of TGF-β1 (1–10 ng/ml), while low concentrations of TGF-β1 (1–10 pg/ml) promoted osteoclast maturation [56], 57].

Interestingly, some reports suggest that BPs may paradoxically enhance osteoclastogenesis via upregulation of interleukin-6 (IL-6) and activation of the STAT3 signaling pathway in MLO-Y4 cells, a well-established osteocyte-like cell line *in vitro*, leading to dose-dependent increased RANKL expression, from a dosage from 0.1 to 1 μM [58]. Although the precise effects of bisphosphonates (BPs) on osteoblasts and osteoclasts remain incompletely understood, their actions appear to follow a dose-dependent, paradoxical pattern: at lower concentrations (typically 10^−9^ to 10^−6^ M), BPs exert a pro-osteoblastogenic effect, whereas at higher concentrations (above 10^−5^ M), an inhibitory influence predominates [59] The stimulatory effects on IL-6/STAT3 signaling were observed at lower, sub-cytotoxic concentrations (e.g., 0.1–1 µM for zoledronic acid in cell culture), which may more closely mimic conditions in the bone microenvironment during chronic therapy. In contrast, the well-known pro-apoptotic and anti-resorptive effects typically require higher(e.g., 50–100 µM), often supra-physiological concentrations in standard *in vitro* assays [58], 60], 61]. These complex and sometimes contradictory effects highlight the multifaceted impact of antiresorptive agents on bone remodeling dynamics. This critical dose-dependent duality underscores a major translational challenge: any biomaterial designed to modulate this pathway must achieve a highly specific local drug concentration, which is difficult to ensure in a dynamic *in vivo* environment. Collectively, these molecular alterations contribute to a net suppression of bone turnover, impaired healing, and necrotic bone retention. Although significant progress has been made in delineating the underlying mechanisms, the precise interplay among these signaling pathways remains incompletely understood and warrants further investigation. Future biomaterial systems could be engineered to modulate specific pathways, such as RANKL signaling or TGF-β release, hereby translating mechanistic insights into targeted therapeutic strategies.

### 2.2 Disturbances of angiogenesis

Impaired angiogenesis is another key contributor to the pathogenesis of MRONJ, usually correlating to delayed wound healing and fistulization. Under normal physiological conditions, angiogenesis plays an essential role in bone repair and regeneration by facilitating nutrient delivery, cell recruitment, and waste removal. Antiresorptive agents, particularly BPs, disrupts neovascularization at sites of injury, delaying tissue repair and promoting osteonecrosis [62], 63].

VEGF, a central mediator of angiogenesis, is directly suppressed by nitrogen-containing BPs such as zoledronic acid (ZA). This downregulatory effect exhibits dose-dependent characteristics and reversibility, which suggests that early prophylaxis against pathological deterioration and the precise calibration of both the timing and duration of therapeutic interventions targeting angiogenesis restoration are critically important [64], 65]. Both *in vitro* and *in vivo* studies have shown that ZA reduces endothelial cell proliferation, migration, and capillary sprouting, thereby attenuating angiogenic responses critical for healing [38]. Additionally, whole exome sequencing showed BPs interfere with TGF-β signaling, a pathway known to stimulate VEGF synthesis, further exacerbating angiogenic deficits [66].

The interdependence between osteogenesis and angiogenesis is especially pronounced during skeletal remodeling. Osteogenesis-angiogenesis coupling represents this spatiotemporally coordinated interdependence between bone formation and vascularization, mediated by bidirectional signaling through direct cellular interactions (e.g., gap junctions) and indirect mechanisms (paracrine/endocrine factors, extracellular vesicles), orchestrated by bone-related cells (osteoblasts, osteoclasts, mesenchymal stem cells) and vascular endothelial cells, which collectively activate intracellular pathways to regulate skeletal development, remodeling, and repair [67]. Preosteoclasts secrete platelet-derived growth factor-BB (PDGF-BB), which coordinates vascularization with bone formation. Experimental evidence indicates that ZA inhibits PDGF-BB release from osteoclasts, thereby impairing angiogenesis and osteogenesis in both bone marrow mesenchymal stem cells (BMMSCs) and endothelial progenitor cells [68]. Based on these findings, Gao et al. have proposed that the early-stage suppression of PDGF-BB-mediated angiogenic in preosteoclast cultivated *in vitro* and osteogenic pathways may represent a central mechanism in MRONJ pathophysiology [69]. This mechanistic insight provides a rationale for developing functionalized biomaterials like VEGF-mimicking peptide scaffolds or PDGF-BB delivery system to precisely restore angiogenesis-osteogenesis coupling in MRONJ lesions.

### 2.3 Dysregulation of inflammation and immune response

Emerging evidence indicates that immune dysfunction and chronic inflammation are integral components of MRONJ pathogenesis [70]. The immune system has bidirectional effect both on bone metabolism and angiogenesis, highlighting the critical need for developing immunomodulatory biomaterials [71], 72]. Among the immune cells involved, macrophages play a central role in regulating bone immunity, and their dysregulated activity has been linked to disease progression. Bisphosphonate exposure, particularly with ZA, has been shown to elevate toll-like receptor 4 expression, suppress anti-inflammatory M2 macrophage polarization, and promote the pro-inflammatory M1 phenotype, both *in vitro* and *in vivo* [73], 74]. This shift in macrophage phenotype contributes to excessive inflammatory cytokine release, impaired bone regeneration, and altered remodeling dynamics.

Further molecular insights reveal that ZA-induced activation of caspase-1 triggers pyroptosis through cleavage of Gasdermin D [75]. Unlike apoptosis, pyroptosis results in plasma membrane rupture and the robust release of pro-inflammatory cytokines (IL-1β and IL-18) in macrophage cultivated *in vitro*, thereby creating a self-perpetuating inflammatory cascade that intensifies tissue injury and MRONJ progression [76]. This inflammatory nature of pyroptosis-characterized by excessive macrophage activation and pro-inflammatory factor production-leads to postponed wound healing and impaired tissue regeneration, making it particularly detrimental in the MRONJ microenvironment where sustained inflammation exacerbates osteonecrosis. ZA also upregulates lysine-specific demethylase 6A/B (KDM6A/B) in macrophages, further reinforcing pro-inflammatory signaling cascades implicated in MRONJ [76]. However, the above results were obtained only through *in vitro* cultivation and there is no evidence from *in vivo* studies yet.

In addition, ZA has been shown to induce endoplasmic reticulum stress (ERS)- related inflammation in macrophages by increasing phosphodiesterase 4B (PDE4B) expression [45]. Pharmacologic inhibition of PDE4B using rolipram has been reported to attenuate MRONJ severity in animal models, highlighting ERS as a potential therapeutic target [45]. However, the current progress of this research is limited to the animal stage, and the mechanism by which ZOL stimulates the upregulation of PDE4B in macrophages remains unclear [45]. Biomaterials designed to modulate oxidative or ER stress pathways (e.g., antioxidant-loaded scaffolds) may offer targeted therapeutic advantages in MRONJ. Elevated IL-17F levels in MRONJ lesions also suggest enhanced Th17 cell activity, indicating that pro-inflammatory T-cell responses may further contribute to disease pathology, and IL-17 inhibition (e.g., anti-IL-17 antibodies) or immunoregulatory biomaterials may represent promising therapeutic strategies, provide a clearer bridge between immunopathology and future material-based interventions [77].

Beyond immune cell dysfunction, impaired lymphangiogenesis also appears to play a role in MRONJ. Lymphatic vessels are critical for maintaining immune homeostasis and facilitating bone repair, and their suppression has been observed in MRONJ models *in vivo*, correlating with prolonged inflammation and delayed healing [45], [69], [70], [71], [72], [73], [74], [75], [76], [77], [78], [79], [80]. ZA induces oxidative stress and autophagy-related cell death in lymphatic endothelial cells (LECs) *in vitro*, which may impair lymphatic remodeling and drainage [81], 82]. Qin et al. demonstrated that ZA disrupts the NAD+/SIRT6/XBP1s signaling axis, impairing SIRT6-mediated autophagy regulation and promoting ERS-induced apoptosis in LECs *in vitro* [83]. The conditional knockout of *SIRT6* impaired lymphatic drainage function by promoting ERS‐induced apoptosis in LECs, thereby exacerbating MRONJ [83]. Cao et al. found zoledronic acid contributes to the pathogenesis of medication-related osteonecrosis of the jaw (MRONJ) by inhibiting the PI3K/AKT signaling pathway in endothelial cells, thereby impairing both angiogenesis and lymphangiogenesis. Exogenous supplementation with insulin-like growth factor-1 (IGF-1) can partially reverse this pathway inhibition and restore the angiogenic and lymphangiogenic capacities of endothelial cells. Importantly, in a rat model of MRONJ, therapeutic intervention targeting this pathway (e.g., via IGF-1 administration) effectively rescued the lymphangiogenic defect and promoted the healing of bone defects. These *in vivo* findings further validate lymphangiogenesis as a reliable therapeutic target for the treatment of MRONJ [84]. These findings suggest that lymphatic dysfunction is an underexplored but potentially important mechanism in MRONJ pathogenesis, warranting further investigation into materials promoting lymphangiogenesis (e.g., VEGF-C delivery platforms) to be explored as adjunctive therapies. To robustly validate lymphangiogenesis as a therapeutic target, future studies employing large-animal models (e.g., porcine or canine) that more closely recapitulate human jaw anatomy and pathophysiology are essential.

### 2.4 Involvement of oral microorganisms

Microbial colonization plays a pivotal role in the pathogenesis of MRONJ. Dental procedures and pre-existing infections can create entry points for microbial infiltration into bone tissue, significantly increasing the risk of osteonecrosis, particularly in individuals with BP-altered bone metabolism. The oral microbiome is composed of diverse bacterial and fungal communities capable of forming resilient biofilms, structured microbial consortia embedded within an extracellular polymeric matrix. These biofilms exhibit enhanced resistance to antimicrobial agents and generate acidic microenvironments that facilitate osteoclast activation and differentiation, further compromising bone integrity [47]. Biofilms can also interfere with surgical debridement and antibiotic penetration, limits the local concentration of the antibiotic, reinforcing why localized, sustained antimicrobial delivery from biomaterials may offer advantages [85].

Microbial dysbiosis, particularly involving shifts in the balance between Gram-positive and Gram-negative bacterial populations, has been associated with MRONJ progression [86]. Saccharolytic bacteria such as Streptococcus spp. have been implicated in the acidification of the local microenvironment, which favors bone resorption and necrosis [87]. However, it remains unclear whether these microbial species are primary initiators or opportunistic colonizers of MRONJ lesions. Given that this distinction critically influences antimicrobial biomaterial design, longitudinal studies tracking microbial dynamics and gnotobiotic studies with controlled colonization models should be prioritized. Gram-negative pathogens also contribute to disease progression by promoting inflammatory signaling and enhancing osteoclastic activity [87], 88]. Notably, the adhesion of *Streptococcus mutans* to necrotic bone, along with the detection of *Aggregatibacter actinomycetemcomitans* and *Porphyromonas gingivalis* in necrotic tissue, supports a significant microbial component in MRONJ pathophysiology [89]. Although the causal role of these microorganisms is still under investigation, their persistent presence underscores the importance of microbial management in MRONJ prevention and treatment.

## 3 Localized biomaterial therapies for MRONJ

### 3.1 Calcium phosphate (CaP) materials

CaP-based biomaterials, such as hydroxyapatite (HA), α- and β-tricalcium phosphate (α-TCP, β-TCP), and biphasic calcium phosphate (BCP), have long demonstrated clinical utility in bone repair due to their compositional similarity to native bone mineral, biodegradability, osteoconductivity, and ability to form stable bone-implant interfaces [90], [91], [92]. Despite these advantages, their relatively low fracture toughness limits their use in load-bearing applications, such as mandibular molar region [93].

Nanostructured, porous CaP materials have garnered attention as drug delivery platforms, offering high surface area, structural stability in physiological environments, and pH-sensitive dissolution, features that support controlled release of therapeutic agents in acidic microenvironments, such as those found in inflamed or necrotic tissue [94]. This is because the acidic conditions promote the dissociation of calcium and phosphate ions from the material’s structure, leading to its dissolution and allowing for the controlled release of drugs encapsulated within or bound to the material. In addition, their ionic degradation products (Ca^2+^ and PO_4_^3−^) enhance drug internalization by cells and mitigate particle accumulation [95]. CaPs are thus widely utilized for the local delivery of pharmaceuticals, bioactive proteins, and genetic materials. However, the functional integrity of sensitive molecules like growth factors must be preserved during loading, as CaP matrices may promote protein denaturation [96]. Modification strategies to preserve protein activity, such as surface coatings, encapsulation, or co-delivery with stabilizers, would provide a pathway for addressing this limitation.

Among CaPs, β-TCP is particularly notable for its resorbability and biocompatibility. Unlike non-degradable HA, β-TCP is broken down via osteoclast-mediated resorption or passive dissolution [97], 98]. Its osteoconductive and osteoinductive characteristics have led to broad clinical use in orthopedic and dental grafting applications [98], [99], [100]. In the context of bisphosphonate therapy, local inflammation and lowered pH facilitate BP release from bone surfaces into adjacent soft tissues [101]. β-TCP exhibits a high binding affinity for BPs due to the P–C–P moiety present in these molecules, making it a promising strategy for localized BP sequestration in MRONJ prevention [102]. Funayama et al. established a MRONJ animal model using 8-week-old male Sprague-Dawley rats [103]. The rats were injected intravenously with ZA at a dose of 1.2 mg/kg per week for 2 weeks, followed by extraction of the left mandibular first molar [103]. In the treatment group, β-TCP was implanted into the extraction socket [103]. The authors found that β-TCP could adsorb bisphosphonates *in vivo*, with a concentration of 1.36 μg/g of ZA detected in the residual β-TCP [103]. In contrast, the ZA concentration in the connective tissue of the extraction socket was 0.61 μg/g in the β-TCP group and 0.79 μg/g in the control group [103]. These results demonstrate that β-TCP placement in extraction sockets adsorbed alveolar bone-derived BPs, reducing peri-tissue drug accumulation and lowering MRONJ risk [103]. This effect was linked to enhanced autophagy modulation, as evidenced by increased expression of autophagy markers (Atg9a, LC-3) and suppression of Rubicon, an autophagy inhibitor, suggesting that autophagy serves as a therapeutic target while β-TCP functions as a potent modulatory biomaterial [103]. However, pure β-TCP suffers from mechanical limitations that may be addressed through pore optimization or composite integration with other structural, like polymers (Polylactic Acid (PLA) and Poly-l-lactic Acid (PLLA)) and metallic materials (Strontium (Sr), Gallium (Ga) and Zinc (Zn) materials) [104].

BCP ceramics, composed of HA and β-TCP in variable ratios, offer a synergistic balance of mechanical integrity and bioresorbability. Adjusting the HA-to-β-TCP ratio enables tailored degradation rates and osteointegration. The interconnected porous network of BCP facilitates angiogenesis, nutrient exchange, and cellular infiltration, supporting osteoblast attachment and extracellular matrix deposition. Beyond its structural role, BCP also functions as a local drug delivery system. Recent studies show that BCP can adsorb ZA locally, mitigating soft tissue damage and promoting bone healing in MRONJ models, with a postoperative wound healing rate of 100 % observed 14 days after surgical intervention. Paulo et al. fabricated a porous bone scaffold comprising 75 % HA and 25 % β-TCP with 80 % porosity and 300–500 µm pores. In a Wistar rat model, this scaffold, applied post-extraction, significantly reduced bisphosphonate uptake in jawbone tissue, as shown by nuclear imaging analysis [105].

Efforts to improve CaP functionality have focused on incorporating osteogenic growth factors such as bone morphogenetic proteins (BMPs), key members of the TGF-β superfamily [31], 106]. BMPs are crucial in maintaining bone homeostasis through dual regulation of resorption and mineralization via specific signaling pathways [32]. BMP-2, the most studied isoform, enhances both osteoblast differentiation and osteoclast activity, positioning it as a powerful therapeutic agent for skeletal regeneration [107], 108]. However, BMP-2 delivery requires an appropriate carrier due to its high diffusibility in aqueous environments, because the burst release of BMP-2 has been associated with ectopic bone formation or inflammation [33], 107], 109]. Studies indicate that a sustained release kinetics lasting from 2 weeks to 42 days can align with the natural timeline of bone regeneration, thereby achieving superior osteogenic outcomes compared to burst release [110]. Regarding concentration, 1.5 mg/mL rhBMP-2 has been identified as the most effective concentration for inducing *de novo* bone formation, exemplified by a dosage regimen of 0.3 mg per socket (i.e., 1.5 mg/mL, 0.2 mL) in alveolar bone applications [111]. Moreover, employing microgram-level low-dose BMP-2 combined with long-term sustained release has been reported to help reduce the risk of adverse effects [110], 112]. Consequently, the optimal BMP-2 controlled-release characteristics should not be regarded as fixed; rather, they ought to be tailored according to the specific surgical indication and the biological profile of the individual patient. Mikai et al. demonstrated that BMP-2-loaded β-TCP significantly enhanced new bone formation and reduced necrosis in murine extraction sockets, supporting its value in MRONJ prevention [107]. Similarly, Dang et al. reported that recombinant human BMP-2 (*Escherichia coli*-derived) loaded onto β-TCP restored osteocyte networks and eliminated microcracks in alveolar bone affected by MRONJ [113].

Importantly, though low concentrations of BMP-2 demonstrate osteoinductive properties and provide protective effects against inflammation in MRONJ, its site-specific and concentration-dependent effects vary with anatomical location and local environment, necessitating precise control of spatial and temporal release [114]. For instance, high concentrations of BMP-2 have been reported to induce substantial adverse effects, including inflammation, bone resorption, tissue swelling, seroma formation, and potential carcinogenicity [115].To address this, novel strategies such as surface modification and controlled-release coatings have been developed [116]. Han et al. engineered a BMP-2-loaded BCP scaffold coated with a multilayer A-PSQ film synthesized from (3-aminopropyl) triethoxysilane via sol-gel chemistry [34]. This modified system achieved sustained BMP-2 release by employing heparin to stabilize and protect the protein from proteolytic degradation, and demonstrated robust osteogenic activity in preclinical models. Despite its promise, surface-functionalized BMP-2/BCP platforms remain underexplored in MRONJ contexts, highlighting the need for future studies to utilize MRONJ-specific animal models or inflammatory microenvironments to further test their efficacy under clinically relevant conditions.

Collectively, these findings underscore the versatility of CaP materials, particularly β-TCP and BCP, as both structural scaffolds and localized delivery vehicles for anti-resorptive agents and regenerative biologics. Integrating programmable BMP-2 release systems into CaP-based constructs holds particular promise for advancing MRONJ prevention and therapy with enhanced precision and efficacy.

### 3.2 Bioactive glass materials

Bioactive glasses (BGs) are a class of multifunctional, biocompatible, and biodegradable inorganic materials widely utilized in orthopedic and dental applications. Several BG-based formulations have already received regulatory approval for clinical use [117], [118], [119], [120]. Based on their primary network-forming oxide, BGs are classified into three categories: silicate-based (SiO_2_), phosphate-based (P_2_O_5_), and borate-based (B_2_O_3_) systems [121]. Among them, B_2_O_3_ glass exhibits a relatively balanced combination of mechanical properties and degradation rate, along with favorable biocompatibility [122]. In contrast, SiO_2_ glass demonstrates excessively slow degradation, while P_2_O_5_ glass suffers from overly rapid degradation and insufficient mechanical strength [122]. When exposed to physiological fluids, BGs undergo surface dissolution, releasing calcium and phosphate ions that facilitate the formation of a hydroxyapatite-like layer. This newly formed layer promotes a robust chemical bond with bone, aiding in tissue regeneration and repair [123].

The bioactivity and ion release profiles of BGs can be precisely tuned by altering the glass network connectivity and incorporating various network modifiers and intermediate ions [123], 124]. The resulting ionic dissolution products have been shown to regulate multiple cellular processes, including proliferation, differentiation, mineralization, and gene expression [117]. Additionally, BGs inherently possess antibacterial activity, which can be further enhanced by doping with metal ions such as Ag^+^, Zn^2+^, Cu^+^/Cu^2+^, Ce^3+^/Ce^4+^, and Ga^3+^, enabling localized infection control in compromised tissue environments [124], 125]. Moreover, BGs exhibit angiogenic properties that support neovascularization, a critical factor in effective bone regeneration and a critical pathological defect in MRONJ [126].

Among BG types, borate-based bioactive glass (BBG) has shown particular promise in the context of MRONJ. Studies have demonstrated that ionic dissolution products from BBG dressings can counteract the detrimental effects of nitrogen-containing bisphosphonates (N-BPs) on osteoclastogenesis and function [127]. BBG is uniquely capable of binding to apatite-adsorbed N-BPs, neutralizing their activity and thereby restoring bone remodeling balance [128]. The underlying chemical mechanism involves a cation-bridged complexation [128]. Divalent cations (e.g., Mg^2+^, Ca^2+^) released from BBG simultaneously coordinate with the phosphonate groups of the N-BPs and the borate anions (B(OH)_4_^−^) in solution, forming stable, insoluble ternary precipitates [128]. This process is driven by the stronger affinity of N-BPs for the borate-cation complex than for the hydroxyapatite bone surface, as supported by binding energy calculations [128]. Consequently, N-BPs are competitively displaced from the bone mineral and sequestered. Owing to their biodegradability, osteoconductivity, and capacity to modulate cellular behavior, BBGs have found expanding applications in bone repair, wound healing, and neural tissue engineering, yet they have not entered the clinical application stage for MRONJ [129].

Su et al. reported that BBG mitigated N-BP-induced inhibition of osteogenesis and angiogenesis, thereby preventing MRONJ development *in vivo*, and this dual-action potential makes BBG a strong candidate for combination strategies with other biomaterials or growth factors [130]. BBG enhanced the proliferation and differentiation of bone marrow mesenchymal stem cells and endothelial cells *in vitro* by binding to N-BPs through direct chemical binding of borate ions to the electron-deficient sites on hydroxyapatite and the formation of insoluble precipitates through complexation between magnesium/borate ions and N-BPs, reducing their cytotoxic effects, and accelerating extraction site healing *in vivo*. These results underscore BBG’s dual role in bone regeneration and localized drug modulation, highlighting its therapeutic potential for MRONJ prevention [130].

Combinatorial approaches further enhance BG efficacy. In preclinical models, filling extraction sockets with F18 bioactive glass combined with pericardial membrane coverage synergistically reduced gingival ulceration, osteolysis, osteomyelitis, bacterial infiltration, and abscess formation [131]. This combination is effective because bioactive glass provides bioactivity, promoting bone regeneration and antibacterial properties, while the pericardial membrane addresses structural limitations, offering a barrier function and mechanical support [131]. This synergy reinforces the rationale for future hybrid strategies that integrate bioactive materials with structural components to optimize therapeutic outcomes.

Despite these advances, balancing the intrinsic bioactivity of BGs with adequate mechanical strength remains a challenge, particularly in load-bearing sites like the posterior mandible and molar-bearing regions [123], 131]. To overcome this, surface engineering techniques, such as molecular grafting to enhance cell signaling and integration, nanocoatings to promote surface bioactivity and protein binding and surface functionalization to improve biocompatibility, have been explored to enhance BG interaction with host tissue and therapeutic efficacy [35], 118], 132], 133]. These surface modifications reflect ongoing innovation aimed at optimizing BG-based platforms for clinical use in MRONJ management.

### 3.3 Hydrogel materials

Hydrogels are three-dimensional, crosslinked polymeric networks capable of absorbing and retaining large volumes of water (>90 %). Their biocompatibility, biodegradability, and tunable osteoconductive and osteoinductive properties have made them increasingly attractive for bone regeneration applications [36]. These hydrogels mimic the native extracellular matrix (ECM), offering mechanical support and promoting cellular functions such as adhesion, proliferation, and differentiation [36], 134]. Their mechanical and physicochemical properties, including porosity and degradation rate, can be modulated through the choice of polymers and crosslinking methods, enabling precise control over material performance and therapeutic payload release [134]. However, the mechanical properties of conventional hydrogels are inferior to those of native bone, posing a challenge for load-bearing applicationsit. It is reported that the elastic modulus of conventional hydrogels typically falls within the kilopascal (kPa) range [135], 136], which is several orders of magnitude lower than that of natural bone tissue in the gigapascal (GPa) range [137]. And the compressive strength of commonly used hydrogels mostly falls within a range of 10–100 kPa, while the compressive strength varies between 100 and 200 MPa for cortical bone, and between 2 and 20 MPa for cancellous bone [135], 136], 138].

Hydrogel porosity and pore interconnectivity critically influence nutrient diffusion, vascularization, and tissue integration. The hydrogel matrix serves as a highly adaptable delivery system capable of encapsulating and releasing a wide range of bioactive agents, including small molecules, proteins, and cells. However, the direct application of bioactive factors *in vivo* is often limited by rapid enzymatic degradation, low local retention, and off-target diffusion, particularly severe in the oral/maxillofacial region due to the presence of salivary enzymes and mechanical stress, which can lead to subtherapeutic efficacy or adverse effects [139]. To address these challenges, hydrogels have emerged as promising vehicles for spatiotemporally controlled delivery systems. Their hydrated polymer networks help confine therapeutic agents within mesh-like structures, enhancing stability and prolonging local bioactivity [140], 141]. Recent advancements in hydrogel design focus on integrating multifunctional therapeutic strategies tailored to the multifactorial pathology of MRONJ, like simultaneously targeting both osteogenesis and inflammation modulation, as elaborated in the next paragraph.

One such approach involves the intelligent delivery of growth factors. Brierly et al. developed a star-shaped poly (ethylene glycol) (starPEG) hydrogel system functionalized with matrix metalloproteinase (MMP)-cleavable linkers and heparin modified with maleimide groups [142]. This system enables dual functionality: heparin binds multiple growth factors, while the MMP-sensitive sequence enables controlled release in response to enzymatic activity, facilitating the clearance of abnormally elevated MMPs in the inflammatory and necrotic bone tissues associated with MRONJ. Incorporation of RGD peptides further enhances cell adhesion and osteogenic differentiation. In a rat MRONJ model, local application of rhBMP-2-loaded starPEG-RGD-heparin hydrogels during tooth extraction increased osteoclast activity, bone volume, and osteocyte density, while mitigating osteonecrosis risk. The hydrogel’s ability to reduce BMP-2 leakage and achieve sustained release in DMEM medium *in vitro* (<10 % over 14 days) represents a significant improvement over conventional collagen sponge-based carriers, matching the initial healing timeline of post-extraction wounds in clinical settings [142].

Hydrogel-based vascular regeneration also offers promise for MRONJ treatment. VEGF, a master regulator of angiogenesis, has been shown to promote neovascularization and accelerate healing in MRONJ-affected tissue. Sharma et al. designed a gelatin-hyaluronic acid hydrogel loaded with heparin-conjugated VEGF to achieve sustained delivery in a ZA-induced maxillary defect model [64]. This system maintained VEGF release for over 28 days in a solution containing 1 mL phosphate buffered saline (PBS) supplemented with Bovine serum albumin (BSA 1 %), heparin (10 μg mL^−1^) and EDTA (1 mM at 37 °C) *in vitro* and significantly upregulated vascular markers such as von Willebrand factor (vWF), VEGF receptor 2 (VEGFR-2), and CD105. It also downregulated inflammatory markers including IL-1β and TNF-α at the mRNA level. Biochemical analyses further confirmed increased peripheral VEGF levels and decreased circulating IL-1α/IL-1β, underscoring the hydrogel’s dual angiogenic and anti-inflammatory effects, directly relating to two central pathophysiological drivers of MRONJ [64].

Despite their clinical promise, current hydrogel systems face several limitations. A key concern is the mismatch between hydrogel degradation rates and post-extraction healing in MRONJ patients healing that may exceed 12 weeks, which may compromise mechanical stability and impair long-term healing [143]. Additionally, many systems struggle with inadequate control over drug release and lack sufficient mechanical strength for use in high-stress environments [144].

To overcome these issues, two advanced hydrogel platforms have been proposed: stimuli-responsive hydrogels and 3D-printed constructs. Stimuli-responsive hydrogels adapt to pathological microenvironments by detecting specific biomarkers, such as pH shifts, reactive oxygen species (ROS), enzymatic activity, and temperature changes, to trigger localized, on-demand drug release, which enables precise alignment of drug activity with inflammation flares or acidic pH events in necrotic zones, which is especially important for temporally heterogeneous conditions like MRONJ [145]. This dynamic behavior enhances therapeutic precision and minimizes systemic toxicity, particularly in inflammatory and oxidative contexts characteristic of MRONJ.

In parallel, 3D-printed hydrogels offer structural and spatial precision through customizable pore architecture, mechanical reinforcement, and hierarchical design mimicking natural bone [146]. The ideal porosity for bone regeneration scaffolds is suggested to be greater than 60 %, with pore sizes exceeding 100 μm [147]. These scaffolds can be embedded with osteoprogenitor cells and cytokines to create bioactive platforms that promote vascularized bone regeneration through synergistic material-cell interactions.

Together, these advances address longstanding challenges in hydrogel design, enabling better control over degradation, mechanical performance, and spatiotemporal delivery. As such, engineered hydrogels represent a next-generation therapeutic strategy for the localized and personalized treatment of MRONJ. However, it is critical to recognize that load-bearing oral tissues, such as the alveolar bone, demand exceptional resistance to masticatory forces and biomechanical stresses, potentially limiting their applicability as structural scaffolds in such demanding environments.

### 3.4 Metallic materials

Metallic biomaterials play a pivotal role in bone repair due to their superior mechanical strength compared to calcium phosphates, natural polymers, and BGs [37]. However, conventional metals such as stainless steel and titanium alloys have notable limitations, including corrosion-related toxicity, lack of osteoinductive properties, and non-degradability, often necessitating secondary surgical removal, which is particularly problematic in maxillofacial surgery, where anatomical access is more restricted than in long bones, adding clinical relevance [41], 148]. To overcome these drawbacks, a new generation of biodegradable metals, including iron (Fe), magnesium (Mg), zinc (Zn), and their respective alloys, has emerged as promising candidates for clinical application in orthopedic and maxillofacial bone repair [37].

Among these, Mg and its alloys have gained substantial attention as revolutionary biodegradable implants. Their favorable mechanical characteristics, including a compressive yield strength and elastic modulus closely matching that of cortical bone, help minimize the stress-shielding effect often seen with traditional metallic implants, facilitating load transfer during mastication, increasing osteogenic stimulus and restore the jaw’s normal structure and function [37], [149], [150], [151]. In physiological environments, Mg degrades through electrochemical reactions with water, forming magnesium hydroxide (Mg(OH)_2_), hydrogen gas (H_2_), and releasing Mg^2+^, hydroxide ions (OH^−^), and other alloying elements [152]. Modern modified magnesium alloys, through alloy design and surface modification, can now control the degradation time within the range of 3–12 months, which basically matches the healing period of 3–6 months [153], 154]. However, it should be noted that excessive accumulation of hydrogen in tissues can cause subcutaneous emphysema, wound dehiscence, and infection, resulting in impaired bone healing [155], 156]. Surface coatings, alloy treatments, etc. have been reported to be effective improvement methods [157]. The release of Mg^2+^ ions contributes to bone healing through multiple mechanisms: it promotes osteoblast activity, modulates osteoclast function, supports angiogenesis, and exhibits anti-inflammatory and antibacterial effects, directly addressing key MRONJ features [41], 150], 158]. These multifunctional properties make magnesium-based materials attractive candidates for counteracting the anti-resorptive and anti-angiogenic disturbances characteristic of MRONJ.

Building on this rationale, Zhu et al. conducted a pioneering study using Mg-based implants by employing a rat model induced with zoledronic acid (130 μg/kg) and dexamethasone (3.8 mg/kg) and creating a penetrating bone defect near the extraction socket of the first mandibular molar [159]. Their results demonstrated that Mg implants significantly reduced osteonecrosis incidence by enhancing vascularization through VEGF and calcitonin gene-related peptide (CGRP)-mediated signaling pathways. These findings support the use of Mg-based devices as internal fixation tools for patients at elevated MRONJ risk.

Despite these promising outcomes, several challenges persist, most notably, the mismatch between degradation rates of metallic implants and the slower pace of bone regeneration [37]. Strategies such as alloying, as well as surface modification techniques including polymer coatings, zwitterionic coatings, and microarc oxidation, have been proposed to fine-tune degradation kinetics, improve biocompatibility, and enhance mechanical resilience [150]. In addition to magnesium, emerging materials like porous tantalum and bismuth alloys have shown potential for bone integration and multifunctionality, although their application in MRONJ-related contexts remains unexplored [37].

Importantly, the exact molecular pathways by which Mg exerts its therapeutic effects in MRONJ remain unclear. Further mechanistic studies, such as those focusing on macrophage polarization and VEGF-R expression, are needed to elucidate how magnesium modulates the osteoimmune and angiogenic environments in MRONJ, which will be essential for optimizing its clinical use [159].

### 3.5 Hybrid materials

One of the enduring challenges in tissue engineering is developing scaffold systems that simultaneously satisfy the often-competing demands of mechanical strength, bioactivity, and biodegradability. Conventional materials frequently present a trade-off: mechanically robust scaffolds, such as metals or ceramics, often lack bioactivity, while bioactive and biodegradable materials, especially those with high porosity, tend to suffer from poor structural integrity, may cause scaffold collapse under masticatory forces or inadequate socket space maintenance. Hybrid materials, which integrate organic polymers with inorganic bioactive components, have emerged as a transformative strategy to overcome these limitations, particularly in complex diseases such as MRONJ. By combining the degradability, flexibility, and biofunctionality of polymers with the mechanical reinforcement and multifunctionality of inorganic nanoparticles, hybrid scaffolds offer tunable properties tailored for tissue-specific applications [160]. These dual-phase constructs are capable of load-bearing while also providing antimicrobial, angiogenic, and osteoinductive cues, key functional requirements for the MRONJ microenvironment, indicating hybrid systems are theoretically suited to address, such as acidic pH, chronic inflammation, or microbial colonization.

Magnesium oxide (MgO) nanoparticles are among the most promising inorganic components for hybrid systems due to their broad-spectrum antibacterial activity, low potential for resistance development, and capacity to promote osteogenesis and angiogenesis through Mg^2+^ ion release. These ions modulate inflammation, enhance cytokine expression, and upregulate CGRP signaling to support vascularized bone regeneration [150], 161]. However, the rapid dissolution of MgO may lead to transient cytotoxicity or local alkalinization, emphasizing the need for precise control over nanoparticle loading and release dynamics to ensure safe and effective therapeutic performance, potentially achievable via coating strategies like core–shell or polymer buffering layers. Core–shell materials are a type of composite micro-nano structure composed of a core and a shell. The core and the shell can be made of the same or different materials to achieve efficient loading and controlled release of bioactive molecules. By precisely regulating the shape of the core, the thickness of the shell layer, and the surface morphology, this structure can effectively optimize drug loading efficiency, release kinetics, and biocompatibility [162]. The polymer buffer layer, through its adjustable chemical composition, thickness and degradation properties, can precisely control the drug release kinetics, effectively avoid burst release and achieve sustained or stimulus-responsive drug administration. This layer also has excellent biocompatibility and structural designability, and can be used as a multifunctional carrier to integrate multiple therapeutic agents, and respond to specific physiological or external stimuli (such as pH, enzymes, light) to achieve on-demand drug release [163]. We compared the advantages and disadvantages of these two regulatory release strategies in MRONJ through a table.

To address these challenges, Guo et al. developed a multifunctional hybrid hydrogel system (PEG-bovine serum albumin (BSA)/a-RGD@MgO NPs, or PBM), incorporating MgO nanoparticles as both structural stabilizers and regulators of gelation kinetics to increase crosslink density and augment the compressibility [164]. In this design, MgO nanoparticles mediate the covalent crosslinking of N-hydroxysuccinimide-functionalized hyperbranched PEG with bioactive proteins, BSA and amino-terminated RGD peptides, via amidation reactions. This configuration enables rapid gelation under hemorrhagic conditions, particularly useful during surgical procedures like tooth extraction in MRONJ-prone patients. Sustained Mg^2+^ release activates Osterix^+^ osteoprogenitor cells and promotes type H vessel formation, thereby simultaneously addressing two critical deficits in MRONJ: (1) it stimulates osteogenesis by activating osteogenic progenitors, countering the suppressive effects of antiresorptive drugs on bone formation, and (2) it compensates for angiogenic inhibition by enhancing type H vessel formation, which restores blood supply, oxygenation, and nutrient delivery to bone tissue while promoting osteoblast differentiation via paracrine signaling (e.g., VEGF, PDGF). This dual action restores the coupling of osteogenesis and angiogenesis, reversing the vascular and metabolic dysfunction central to MRONJ pathophysiology. Moreover, the hydrogel exhibits immunomodulatory effects by downregulating pro-inflammatory cytokines (e.g., TNF-α, IL-6) and demonstrates antimicrobial activity through microbial community modulation in female SD rat model. These multifunctional capabilities make the PBM hydrogel a compelling candidate for minimally invasive MRONJ therapy, addressing vascular disruption, inflammation, and infection simultaneously. Nonetheless, further optimization is needed to align degradation kinetics with the pace of bone healing and to fine-tune Mg^2+^ release to avoid cytotoxicity [164].

Mesoporous silica nanoparticles (MSNs) also offer significant potential in hybrid scaffold systems due to their high surface area, tunable porosity, and versatile surface chemistry [39], 165]. MSNs can be engineered to encapsulate and release bioactive agents in response to environmental triggers such as pH, enzymatic activity, or redox conditions. Their surface can be functionalized for ligand targeting or co-delivery of multiple therapeutic agents. However, their lack of mechanical strength and spatial architecture limits standalone use in load-bearing applications, necessitating their integration into supportive 3D scaffolds such as ceramics or biodegradable metals for effective tissue engineering applications [39], 165].

Thavornyutikarn et al. exemplified this strategy by engineering a dual-drug delivery platform combining two types of MSNs, plasma-treated particles loaded with hydrophilic clindamycin (CDM) and amine-functionalized MSNs loaded with hydrophobic geranylgeraniol (GGOH). These nanoparticles were embedded into a carboxymethyl chitosan (CMCS) hydrogel matrix, forming a biphasic composite system [143]. The resulting hydrogel sustained antibacterial activity against *Streptococcus sanguinis* for 14 days and released both drugs over a nine-day period. *In vitro* studies confirmed cytoprotective effects for osteoblast and osteoclast progenitor cells exposed to ZA, positioning this system as a promising prototype for localized MRONJ therapy. While preclinical efficacy remains to be validated *in vivo*, these findings support the utility of nanocarrier–hydrogel hybrid platforms for MRONJ treatment. To establish the clinical translational potential of this therapeutic system, future *in vivo* research is suggested to quantify relevant endpoints, encompassing bone volume assessments, angiogenesis quantification, wound healing evaluations, and inflammatory biomarker analysis.

Hybrid materials offer a powerful framework for designing next-generation scaffolds that simultaneously address the mechanical, biological, and pharmacological demands of MRONJ management. It is plausible that patient subgroups like high-risk groups (e.g., cancer patients on long-term IV bisphosphonates) might particularly benefit. The convergence of nanotechnology and biomaterials engineering allows for highly customizable, multifunctional platforms. Future research should emphasize patient-specific designs and programmable systems that dynamically adapt to MRONJ’s complex and multifactorial pathology. Table 2 summarizes the advantages and disadvantages of drug release control strategies in MRONJ [162], 163], [166], [167], [168].

**Table 2:** Pros/cons of release tuning strategies in MRONJ.

		Pros	Cons	Ref
Core-shell	Release	Responsive release combinatorial therapies, sustainable release, good drug loading efficiency	Complex drug release kinetics increased nanoparticle size higher reticuloendothelial system clearance	[167], 169], 170]
	Cytotoxicity	Good biocompatibility, low immunogenicity	Toxicity risk of inorganic materials	
	Mechanical support	Adjustable mechanical strength		
Polymer buffering	Release	Responsive release, sustainable release	Relatively slow release speed	[168], 171]
	Cytotoxicity	Good biocompatibility, low immunogenicity	Risk of acidic degradation products	
	Mechanical support	Adjustable mechanical strength		

### 3.6 Platelet concentrates

Platelet concentrates (PCs) are autologous blood-derived products enriched with supraphysiological levels of platelets and growth factors. For example, VEGF enhances angiogenesis while PDGF facilitates tissue repair, counteracting MRONJ-associated angiogenic defects and healing deficits. They are widely used in dental and regenerative medicine for their capacity to enhance tissue repair. Two principal generations of PCs have been developed: first-generation platelet-rich plasma (PRP) and second-generation platelet-rich fibrin (PRF). PRP and its subtype, pure PRP (P-PRP), are derived from anticoagulated blood, whereas leucocyte- and PRF (L-PRF) is obtained from whole blood without anticoagulants [40]. The absence of anticoagulants in PRF preparation allows fibrinogen polymerization under physiological thrombin levels, yielding a more elastic and stable fibrin network compared to PRP’s rigid structure (induced by anticoagulants), which enhanced structural flexibility facilitates superior cell recruitment, growth factor sequestration, and tissue regeneration, explaining its preferential use over PRP in clinical applications.

The therapeutic rationale for PRP lies in delivering concentrated platelets to sites of injury, where they release bioactive molecules that initiate hemostasis, stimulate connective tissue formation, and support angiogenesis [172], 173]. However, the use of anticoagulants in PRP formulations has been reported to potentially provoke immune responses or coagulopathies, which could limit their safety in some clinical contexts [172].

In contrast, PRF offers a safer and simpler alternative, as it is prepared without anticoagulants and contains platelets, leukocytes, fibrin, and essential growth factors such as platelet-derived growth factor (PDGF), TGF-β1, and VEGF [169]. PRF has shown significant promise in the management of MRONJ by promoting cell migration, proliferation, collagen synthesis, angiogenesis, and immune modulation [169], 170]. These fibrin-rich scaffolds serve three key roles: they provide mechanical support, deliver viable cells, and enable sustained release of growth factors. The growth factors in PRF are rapidly released in the first 2 days after activation, and the release rate gradually slows down in the subsequent time period, but can be released for about 7–10 days, thereby offering critical temporal insights for optimizing clinical applications [171], 174]. Clinical studies consistently report the beneficial adjuvant role of PRF in both prevention and treatment of MRONJ, particularly in accelerating wound healing and bone repair [175], [176], [177], [178]. A prospective controlled clinical study of 85 patients undergoing tooth extraction under chronic anti-resorptive/anti-angiogenic therapy with 8-week follow-up demonstrated significantly higher healing rates in the PRF group (96 %) compared to controls (64.29 %, *p* < 0.01), supporting PRF’s efficacy in preventing MRONJ [179]. However, this study has some shortcomings in its design. Since the study classified patients who refused to use the new treatment as the control group and those who agreed as the experimental group, there is a certain selection bias [179]. This study is also limited by the small sample size and the lack of long-term follow-up to confirm the absence of local recurrences over time [179]. Further randomized controlled studies are necessary.

Notably, the combination of PRF with osteogenic agents such as BMP-2 has demonstrated synergistic effects, outperforming PRF monotherapy in MRONJ models [42]. However, PRF’s clinical utility is limited by its rapid degradation and instability in liquid environments. To overcome these challenges, Tao et al. embedded PRF into a methacrylated gelatin (GelMA)/methacrylated heparin (HepMA) hydrogel and the incorporation of HepMA increased the stability of the crosslinking system and prolonged the degradation time of the hydrogel composite [174]. This system utilized UV-induced covalent crosslinking to enhance mechanical stability and controlled release of growth factors for more than four weeks. The resulting Hep/GelMA-PRF hydrogel scaffold significantly improved osteogenic differentiation, exerted anti-inflammatory effects, and stabilized the local microenvironment in MRONJ models [174].

PRF also serves as a biologically active fibrin scaffold capable of supporting diverse therapeutic components, including stem cells and nanoparticles. Fibrin alone or in combination with other biomaterials has demonstrated utility in promoting the regeneration of bone, nerves, and soft tissue from general tissue healing studies, though the cited models don’t include MRONJ-relevant conditions (e.g., bisphosphonate treatment or socket extraction) [180]. Various formulations within the PRF family, such as PRF, L-PRF, fibrin glue, and fibrin-based hydrogels, have been explored for tissue engineering purposes.

In an innovative clinical approach, Bouland et al. combined autologous adipose tissue-derived stromal vascular fraction (AT-SVF) with L-PRF to treat MRONJ surgical wounds. AT-SVF, which contains mesenchymal stromal cells (MSCs) and endothelial progenitor cells (EPCs), contributes to osteogenesis and neovascularization. L-PRF, in turn, functions as a three-dimensional scaffold, supporting cell viability and sustained growth factor release. The combined therapy effectively promoted both soft tissue healing and bone regeneration in MRONJ patients, suggesting a promising strategy for advanced, personalized care, though the osteogenic mechanism of AT-SVF materials remains to be elucidated [181].

### 3.7 Extracellular vesicles

Stem cell-based therapies have shown considerable promise in the treatment of MRONJ [182]. However, the clinical application of cellular approaches is often constrained by safety concerns, including the risks of thromboembolism, tumorigenicity, and immunogenic responses [183]. As a cell-free alternative, extracellular vesicles (EVs), comprising exosomes, ectosomes, microvesicles, and apoptotic bodies, have emerged as a novel and potent therapeutic modality, due to their osteogenesis and angiogenesis ability, and potent anti-inflammatory and immunomodulatory effects that counteract known pathogenic mechanisms of MRONJ. These lipid bilayer-enclosed particles are secreted by nearly all cell types and possess inherent biocompatibility, low immunogenicity, and robust functional versatility [184], 185].

EVs serve as natural carriers of proteins, lipids, and nucleic acids, enabling them to modulate critical cellular processes such as osteogenesis, angiogenesis, and immune regulation [184], [185], [186]. Stem cell-derived EVs, in particular, offer a lower-risk alternative to whole-cell therapies, while retaining their regenerative and immunomodulatory capabilities. For instance, Huang et al. reported that small EVs derived from adipose tissue (sEV-AT) significantly reduced MRONJ-like lesions in rat model by enhancing angiogenesis and tissue regeneration through the delivery of pro-healing microRNAs and growth factors, increasing number of osteoclasts, the production of collagen fibers and blood vessels and elevating BV/TV, Tb·N, Tb·Th, and decreased Tb.Sp (*p* < 0.001) [187], 188]. Similarly, Watanabe et al. demonstrated that mesenchymal stromal cell-derived EVs (MSC-EVs) reversed ZA-induced osteoblast senescence and inflammatory cytokine production, thereby restoring bone-forming activity and tissue homeostasis [189].

Exosomes, a well-characterized subset of EVs (30–130 nm in diameter), have garnered particular interest for localized MRONJ therapy due to their cargo-carrying capacity and immuno-evasive properties, as they possibly reduce local inflammation and immune reactions at the MRONJ site, creating a favorable microenvironment for bone healing [46]. As lipid bilayer nanocarriers, exosomes facilitate intercellular communication by delivering bioactive molecules that modulate gene expression, signaling pathways, and functional phenotypes in recipient cells. Zheng et al. showed that adipose-derived MSC exosomes suppressed M1 macrophage pyroptosis, a process triggering inflammatory cascades and impairing tooth socket healing, via inhibition of the NF-κB/NLRP3/IL-1β axis, and promoted bone and soft tissue regeneration through IL-1 receptor antagonist (IL-1RA)-mediated anti-inflammatory signaling [75], 184].

Beyond their intrinsic regenerative properties, exosomes offer unique advantages as drug delivery vehicles. Their endogenous origin endows them with excellent biocompatibility, low immunogenicity, and the ability to cross biological barriers, including mucosal and osseous interfaces, which has been proved to accelerate osteogenesis in a mouse calvarial bone defect model, though this trans-barrier capability has not been validated in MRONJ-relevant oral/maxillofacial models [43], [190], [191], [192]. Exosomes also inherit cell-specific targeting capabilities from their parental cells, enabling site-specific delivery of therapeutic cargoes while shielding them from enzymatic degradation.

Exosomes are naturally enriched with regulatory nucleic acids, including DNA, microRNA (miRNA), long non-coding RNA (lncRNA), circular RNA (circRNA), messenger RNA (mRNA), and transfer RNA (tRNA). These molecules regulate gene expression at transcriptional and post-transcriptional levels, influencing recipient cell behavior [44]. miR-149-5p is a miRNA broadly associated with bone metabolism and not specific in MRONJ, which can regulate osteoblast differentiation and bone formation (e.g., by targeting TGFβ2) and modulate inflammatory responses (e.g., by inhibiting IL-6) in bone metabolic diseases and participate in osteoarthritis and rheumatoid arthritis [193]. The role of miR-149-5p in MRONJ pathogenesis is supported by emerging clinical evidence. A clinical study including five multiple myeloma patients with MRONJ and five healthy patients showed miR-149-5p is significantly overexpressed (8-fold) in peripheral lymphoid of patients with MRONJ compared to healthy controls [194]. Shen et al. identified that zoledronic acid-induced macrophages suppress type H vessel formation and osteoclast differentiation via NF-κB-mediated upregulation of miR-149-5p [195]. Their study further demonstrated that local delivery of chemically modified antisense oligonucleotides targeting miR-149-5p significantly improved mandibular healing and angiogenesis, suggesting that miRNA-targeted therapies may provide a novel route for restoring skeletal homeostasis in MRONJ [195].

Despite their therapeutic potential, several hurdles remain before exosome-based therapies can be translated into clinical practice. A key concern is the intrinsic heterogeneity of exosomes, which varies depending on the physiological state and origin of the donor cells, leading to distinct sets of miRNAs and surface ligands and varying tissue affinities and immunogenicity, resulting in uncontrollable activation of signaling pathways or immune inflammatory responses and thus variations between different batches of exosome-based therapeutics, affecting the reliability and reproducibility of treatment outcomes [43]. In addition, technical challenges persist in scaling up exosome production while maintaining consistency in purity, stability, and yield across manufacturing batches, which are mainly issues largely tied to limitations in purification methods (e.g., ultracentrifugation efficiency), bioreactor design (e.g., shear stress effects), and preservation logistics (e.g., cold chain requirements) [190]. Current lab-scale isolation methods, such as ultracentrifugation, are low-yield, time-consuming, and prone to contamination, making them unsuitable for clinical-grade manufacturing. While methods like tangential flow filtration (TFF) offer better scalability and yield, achieving high-purity separation at an industrial scale is still evolving [196]. Current good manufacturing practice compliance represents the foundational milestone, requiring standardized protocols for cell culture, exosome isolation, and quality control to ensure batch-to-batch consistency [197]. For clinical translation, establishment of standardized quality control criteria constitutes a critical milestone, which must define identity, strength, and purity attributes regardless of originator cell type or culture conditions [198], 199]. Standardized characterization metrics must be established, including nanoparticle tracking analysis for concentration and size distribution, transmission electron microscopy for morphology verification, and proteomic profiling of exosomal markers alongside absence of contaminating cellular components [196], 200]. Addressing these milestones through interdisciplinary collaboration is fundamental to transforming exosome therapy from a promising preclinical concept into a reproducible and commercially viable treatment for MRONJ. Moreover, to address these issues, recent strategies also propose the use of patient-specific autologous exosomes as personalized, biocompatible delivery systems, because autologous EVs may better reflect the recipient’s immune and regenerative profile, potentially minimizing rejection or off-target effects, autologous EVs are more viable and impactful in damaged tissues though these approaches require further preclinical and clinical validation [190], 201].

## 4 Systemic biomaterial therapies for MRONJ

Systemic drug delivery has been investigated as a preventive strategy for MRONJ; however, it is hindered by several inherent limitations. Traditional systemic treatments, including antibiotics, anti-inflammatory agents, and bone-modulating drugs, frequently suffer from inadequate drug concentrations at the jawbone, poor tissue specificity, and systemic toxicity due to widespread, non-selective biodistribution. This phenomenon can be attributed to the mandible’s unique vascular anatomy, where osseous perfusion is predominantly derived from the inferior alveolar artery, coupled with its dense cortical bone structure and intricate trabecular architecture that collectively affected therapeutic agent diffusion into the osseous lesions [202]. These challenges compromise both efficacy and safety, as systemically administered drugs often fail to achieve therapeutic levels in the jaw while accumulating undesirably in off-target organs.

To address these limitations, recent studies have proposed innovative biomaterial-based approaches to enhance the efficiency and targeting of systemically administered therapies. One key pathological factor implicated in MRONJ is lymphatic dysfunction induced by ERS, which is triggered by ZA via activation of the NAD^+^/SIRT6/XBP1s signaling pathway. This activation leads to ERS and apoptosis in LECs, exacerbating local inflammation and contributing to disease progression [83]. To mitigate this effect, a novel nanoparticle formulation, ZA-1,2-distearoyl-sn-glycero-3-phosphoethanolamine-polyethylene glycol-rapamycin (ZDPR), was developed to co-deliver ZA and rapamycin. The ZDPR conjugate successfully attenuated ERS-induced apoptosis through mTOR pathway inhibition, exclusively observed in lymphatic endothelial cells so far, improved lymphatic drainage, and alleviated MRONJ symptoms in preclinical models [83].

In parallel, tetrahedral framework nucleic acids (tFNAs) have emerged as a promising class of DNA-based nanostructures for systemic therapy. These three-dimensional nanomaterials exhibit high structural stability, efficient cellular uptake, excellent tissue penetration, and programmable functionality, making them highly versatile platforms in biomedical applications [203]. Cui et al. demonstrated that systemically administered tFNAs counteract the inhibitory effects of ZA on osteoclast differentiation and maturation through direct effect of activating Wnt signaling pathway and reversing the inhibited expression of C-fos and NFATc1 in osteoclasts, highlighting their prophylactic potential against [204]. tFNAs are also well-suited for drug delivery, offering capabilities such as nucleic acid hybridization, physical encapsulation of small molecules, and site-specific functionalization for the delivery of nucleic acids and therapeutic agents [205].

To enhance angiogenesis, a key therapeutic target in MRONJ, a novel nanoparticle was developed by integrating the angiogenic peptide KLT into tFNA scaffolds. The resulting tFNA-KLT construct exhibited high biocompatibility, robust structural integrity, and ease of synthesis [206]. Functionally, tFNA-KLT significantly promoted endothelial cell proliferation, migration, and tubulogenesis *in vitro*, while enhancing vascularization and demonstrating preventative efficacy *in vivo* with a high soft tissue healing rate of 92 % and BV/TV of 0.99, establishing its potential as a systemic pro-angiogenic therapy for MRONJ [206]. However, quantitative pharmacokinetic data for this formulation, such as its circulation half-life or the precise percentage accumulated in the mandibular bone, are currently unavailable in the literature. Future studies are required to define these critical parameters.

Despite these advances, major challenges remain. These include insufficient drug accumulation in the jawbone due to the anatomical characteristics of poor perfusion and thick cortical bone barrier, and deficient bone-homing ligands, lack of standardized dosage protocols, and limited bone-targeting capabilities of current delivery platforms. A key translational gap is the lack of quantitative targeting efficiency metrics (e.g., %ID/g in jawbone, circulation half-life). Establishing these pharmacokinetic and biodistribution profiles is an essential next step to evaluate its clinical feasibility and optimize dosing regimens. Consequently, while systemic administration may be suitable for patients with concurrent systemic skeletal diseases, its utility in MRONJ prevention remains constrained due to the localized pathology may be poorly addressed by systemic bioavailability alone, hence reinforcing the need for site-targeted interventions. The clinical translation of systemic nanoparticle administration for MRONJ treatment necessitates rigorous evaluation of immunotoxicity and mononuclear phagocyte system (MPS) interactions [207]. Upon intravenous injection, nanoparticles encounter the MPS as the primary biological barrier, where Kupffer cells in the liver and splenic macrophages can cause a rapid uptake not only compromises therapeutic efficacy but also raises concerns regarding long-term organ toxicity [207]. To de-risk clinical translation, preclinical studies must incorporate quantitative safety endpoints that extend beyond therapeutic efficacy. Essential safety assessments should include: organ-level biodistribution (e.g., mandible vs. liver/spleen accumulation), pharmacokinetic profiling and clearance kinetics (e.g., half-life, elimination routes), and the measurement of systemic toxicity markers (e.g., plasma cytokines, hepatic/renal injury biomarkers) [48], 208], 209]. Future research should prioritize the development of multifunctional, bone-targeted, and patient-specific delivery systems to reduce systemic toxicity and improve therapeutic outcomes. Tailoring systemic biomaterial strategies to address the unique anatomical and pathological features of MRONJ will be essential for maximizing safety and efficacy.

## 5 Discussion

The integration of advanced biomaterials into the prevention and treatment of MRONJ offers a promising alternative in managing this multifactorial and clinically challenging condition. By specifically targeting key pathogenic mechanisms, such as impaired bone remodeling, angiogenic deficits, inflammatory dysregulation, and microbial imbalance, biomaterial-based strategies offer significant advantages over conventional therapies, which often fall short in efficacy, specificity, or safety.

Recent developments underscore the potential of hybrid and multifunctional systems that combine organic and inorganic components to enable precise control over drug delivery and tissue regeneration, based on direct comparisons with conventional biomaterials, such as β-TCP and HA. For example, β-TCP/Mg-based composites have shown enhanced osteoconductivity and pro-angiogenic effects in preclinical studies by modulating alkaline phosphatase activity and bone sialoprotein expression, possibly involving both direct effects of Mg^2+^ on promoting the proliferation and differentiation of osteoblasts and its downstream signaling pathways such as PI3K pathway [158], 210]. However, achieving a stable balance among mechanical strength, biodegradability, and biological activity remains a key challenge. BG, for instance, effectively sequesters BPs and restores bone homeostasis but lacks sufficient mechanical integrity for application in load-bearing sites [130]. Composite material and coating strategies, such as a BCP coated with a Sr-, Mg- and Zn-doped sol-gel derived BG which show improved mechanical properties, have been attempted to overcome this limitation [49]. Likewise, hydrogels loaded with growth factors such as VEGF or BMP-2 exhibit potent regenerative effects but degrade too rapidly to support sustained tissue integration [64], 142]. The incorporation of multimodal therapeutic strategies with biomaterials is currently under investigation, such as the combined application of beta tricalcium phosphate and antimicrobial photodynamic therapy, and the potential of integrated management systems with combined clinical strategies pairing biomaterials with other treatments method is only beginning to be realized [211].

A persistent challenge in MRONJ biomaterial therapy is material retention within the dynamic and complex oral environment. Salivary flow, microbial activity, and continuous masticatory forces create conditions that compromise the stability and efficacy of locally applied therapies. Inorganic scaffolds, although structurally robust, often fail to conform the complex geometry and achieve seamless tissue integration in irregular bone defects like extraction sockets, thereby affecting tissue integration [103]. In contrast, hydrogel-based materials conform well to defect morphology but are easily displaced due to their hydrophilic swelling behavior and weak mechanical properties, which further diminish under moist conditions [144]. This undermines the retention of therapeutic agents and diminishes localized efficacy. Future research must focus on enhancing the bioadhesive properties of scaffolds through surface modifications, such as the incorporation of catechol groups, bone-specific ligands, or fibrin-based adhesives, to improve interfacial bonding, molecular adhesion, and physical anchorage in wet tissues, though their efficacy remains unvalidated in MRONJ-related *in vivo* models and remain theoretical at this stage [212].

While promising results have been demonstrated in rodent models (Table 3), the translation of these findings to clinical application is limited by several factors [83], 142], 143]. Current preclinical studies predominantly rely on standardized protocols involving ZA administration followed by tooth extraction; however, inconsistency in drug type, dosage, and timing across studies hinders comparability and reproducibility [213], 214]. Moreover, the anatomical and physiological differences in jawbone healing between rodents and humans present a barrier to accurate extrapolation [215]. To bridge this gap, future studies should incorporate large-animal models, such as canine or porcine systems, which better replicate human jawbone structure, mineral concentration, vascular parameters and immune response, and healing kinetics in new bone formation rate of 1.2–1.5 μm/day [216], 217]. Additionally, new model designs incorporating comorbidities and multifactorial variables, such as MRONJ induction by periodontitis, will be essential for simulating clinically relevant MRONJ scenarios [218].

**Table 3:** Collection of MRONJ rodent model establishment methods.

Species	Age	Sex	Drug administration strategy	Interval time and method	Ref.
SD rat	8 Weeks	Male	ZA, 0.06 mg/kg, intravenously, once a week for 2 week	Two weeks after the last administration, extract the left mandibular 1st molar and create bone defects with a size of 2 mm × 3 mm × 2 mm	[31]
Wistar rat	16–18 weeks	Female	ZA, 0.1 mg/kg, intraperitoneally, three times a week for 7 weeks	In the fourth week, extract the left mandibular 1st molar	[32]
C57BL/6J mouse	8–12 weeks	Female	ZA, 0.05 mg/kg, subcutaneously and CY, 100 mg/kg intraperitoneally, twice a week for 3 weeks and 2 more weeks after tooth extraction	In the fourth week, extract the maxillary 1st molars	[33]
C57BL/6J mouse	8–12 weeks	Female	ZA, 0.05 mg/kg, subcutaneously and CY, 100 mg/kg intraperitoneally, twice a week for 3 weeks and 2 more weeks after tooth extraction	In the fourth week, extract the maxillary 1st molars	[34]
SD rat	4 weeks	Female	ZA, 0.125 mg/kg, intraperitoneally twice a week and DEX, 5 mg/kg, intraperitoneally, once a week for 4 weeks	In the fifth week, extract the right mandibular 1st molar	[35]
Wistar rat	9 weeks	Male	ZA, 0.2 mg/kg, intraperitoneally, twice a week for 4 weeks	In the fifth week, extract the left mandibular 1st and 2nd molars	[36]
SD rat	63–67 days	/	ZA, 0.1 mg/kg, intravenously, once a week in week 2 and in week 5 respectively	In the fifth week, extract the right mandibular 1st molar and create bone defects with a size of 3 mm × 2.5 mm × 2 mm	[219]
SD rat	10–12 weeks	Female	ZA, 0.198 mg/kg, intraperitoneally, once a week for 8 weeks	In the fifth week, extract a random side maxillary 1st and 2nd molars and create bone defects with a diameter of 5 mm	[38]
SD rat	8 weeks	Male	ZA,0.13 mg/kg, intraperitoneally, once a week and DEX, 3.8 mg/kg, intraperitoneally, once a week for 5 weeks	In the sixth week, create a penetrating mandibular tunnel with a diameter of 1.3 mm around the first molar root apex and 1 mm wide bone defect below the tunnel	[39]
SD rat	12 weeks	Female	ZA, 0.3 mg/kg, intraperitoneally, once a week for 12 weeks	In the twelfth week, extract the molars and create a penetrating mandibular defect with a diameter of 3 mm	[144]
SD rat	8 weeks	Female	ZA, 0.125 mg/kg, intraperitoneally, twice a week and DEX, 5 mg/kg dexamethasone once a week for 6 weeks	In the seventh week, extract the 1st mandibular molars	[220]
SD rat	8 weeks	Female	ZA, 0.066 mg/kg, intravenously and Dex, 5 mg/kg, intravenously three times a week for 4 weeks	In the third week, extract the left maxillary 1st molar	[40]
C57BL/6N mouse	6–8 weeks	Male	ZA, 1 mg/kg, intraperitoneally, once a day for 4 weeks	In the third week, extract the maxillary first molars	[46]
C57BL/6J mouse	8 weeks	Male	ZA 0.125 mg/kg, intravenously, twice a week for 5 weeks	In the second week, extract the right maxillary 1st molar	[201]
C57BL/6N mouse	8 weeks	/	ZA, 0.125 mg/kg, intravenously, twice a week for 5 weeks	In the second week, extract the right maxillary 1st molar	[83]
Wistar rat	8 weeks	Male	ZA, 0.125 mg/kg, intraperitoneally, twice a week and DEX, 5 mg/kg, intraperitoneally, once a week for 5 weeks	In the second week, extract the first molar of the left maxillary	[48]
SD rat	8 weeks	Male	ZA, 0.125 mg/kg intravenously twice a week and DEX, 5 mg/kg intraperitoneally once a week for 5 weeks	In the fifth weekend, extract the left maxillary first molar at the fifth weekend	[49]

ZA, zoledronic acid/zoledronic acid; CY, cyclophosphamide; DEX, dexamethasone.

Given the heterogeneous nature of MRONJ, driven by differences in drug class, exposure duration, and patient-specific risk factors, personalized biomaterial strategies are urgently needed. For example, patient-specific factors like diabetes or prior infection further demand tailored surface chemistries or antimicrobial coatings to optimize healing outcomes. PRF-based autologous scaffolds, enriched with patient-derived growth factors and stem cells, offer one such personalized solution that enhances healing while minimizing immunogenicity. Similarly, nanoparticle-mediated displacement therapies using low-potency BPs may selectively mitigate pre-adsorbed N-BP toxicity without systemic side effects. Future directions should focus on developing combinatorial biomaterial platforms incorporating EVs for immune modulation, antimicrobial peptides for infection control, and embedded biosensors for real-time monitoring of wound healing dynamics.

The treatment strategies for MRONJ using biomaterials face complex regulatory classification challenges on the path of translational medicine. According to the regulatory framework of the US FDA, bone regeneration materials are classified into different categories based on their composition and risk level: simple synthetic bone substitutes (such as β-tricalcium phosphate, hydroxyapatite) are typically classified as Class II medical devices and can apply for market approval through the 510(k) pathway, demonstrating their substantial equivalence to approved products [219], 220]. However, when the biomaterials contain bioactive factors (such as recombinant human bone morphogenetic protein-2, rhBMP-2) or living cell components, the regulatory path undergoes a fundamental change and may be classified as Class III medical devices or combination products, requiring the submission of strict clinical trial data to prove safety and efficacy through pre-market approval (PMA) or innovative medical device (IDE) pathways [219]. For the specific indication of MRONJ, there is currently no specific biomaterial that has received regulatory approval for this condition. The existing products are mostly used off-label [219]. This regulatory gap highlights the urgency of establishing specific clinical evaluation criteria for MRONJ. Given the multi-factor nature of the MRONJ pathological mechanism (including bone metabolism inhibition, angiogenesis disorder, and immune imbalance), the design of future biomaterials should integrate multi-functional therapeutic strategies, such as a composite system that simultaneously incorporates BPs adsorbents, angiogenic factors, and immunomodulators [220]. Therefore, early scientific consultation with regulatory agencies to clarify the ATMP classification or combination product attributes of the product is crucial for reducing the transformation risk and optimizing the clinical development path.

Plan for Subsequent Experimental Steps. 1) Establish and Validate Standardized Preclinical Models: Develop and disseminate consensus protocols for MRONJ induction in large-animal models (e.g., porcine, canine) that more accurately replicate human jaw anatomy, bone density, and healing kinetics; Standardize key variables across studies, including the type, dose, timing, and route of anti-resorptive/anti-angiogenic drug administration, as well as the method for creating the osseous defect (e.g., tooth extraction). Conduct Head-to-Head Comparative Efficacy Studies: Systematically compare leading biomaterial platforms (e.g., functionalized β-TCP vs. bioactive glass vs. advanced hydrogels vs. hybrid systems) within the same standardized large-animal model; Define primary and secondary endpoints for comparison, such as bone volume/tissue volume (BV/TV), angiogenesis/lymphangiogenesis metrics, inflammatory biomarker reduction, rates of complete mucosal healing, and absence of necrotic bone. 3) Optimize Material Retention and Biointegration: Design and test strategies to enhance scaffold retention in the dynamic oral environment. This includes evaluating surface modifications (e.g., catechol groups, bone-targeting ligands) and bioadhesive formulations to improve anchorage to wet tissue and bone; Investigate methods to better align scaffold degradation profiles with the prolonged healing timeline (often >12 weeks) in MRONJ patients to ensure sustained mechanical support and drug release. 4) Develop and Test Multifunctional, Patient-Tailored Platforms: Engineer and validate “combinatorial” biomaterials that simultaneously address multiple MRONJ pathophysiological factors (e.g., integrating BP-sequestering agents, osteogenic/angiogenic factors, and immunomodulators like exosomes or antimicrobial peptides into a single scaffold); Explore the feasibility of patient-specific designs, such as 3D-printed scaffolds tailored to individual defect morphology or the use of autologous components (e.g., PRF, adipose-derived stromal cells). 5) Define Pharmacokinetic and Safety Profiles for Systemic Nanotherapies: For systemically administered biomaterial therapies (e.g., nanoparticles, tFNAs), conduct rigorous pharmacokinetic studies to quantify mandibular accumulation (%ID/g), circulation half-life, and biodistribution to off-target organs; Perform comprehensive safety assessments, including evaluation of interactions with the mononuclear phagocyte system (MPS) and long-term organ toxicity, to de-risk clinical translation.

## 6 Conclusions

Innovative biomaterial-based strategies hold significant promise in the prevention and management of MRONJ by targeting its underlying pathophysiological mechanisms. Preclinical studies have demonstrated that emerging biomaterials can effectively sequester BPs, restore osteo-angiogenic balance, and modulate inflammatory and immune responses. Despite these advances, several barriers to clinical translation remain. These include insufficient material retention in the dynamic oral environment, poor alignment between scaffold degradation and bone healing kinetics, and inadequate mechanical properties for use in load-bearing sites.

Future developments should prioritize the design of multifunctional biomaterials featuring controlled and stimuli-responsive drug release triggered by pH, enzymes, ROS, enhanced mechanical performance, and customizable architectures tailored to individual patient needs. In parallel, the lack of standardized preclinical models continues to hinder comparative evaluation and regulatory approval, underscoring the need for consistent, reproducible platforms for testing. While biomaterial-based interventions represent a promising therapeutic direction, further research is essential to rigorously assess their efficacy, biosafety, and translational feasibility in clinical settings, especially randomized controlled trials and large-animal validation. We recommend that the scientific and clinical communities establish a consensus-based preclinical testing standard for MRONJ by the year 2030, to harmonize research efforts and accelerate translational progress.

## References

[1] Marx RE (2003). Pamidronate (Aredia) and zoledronate (Zometa) induced avascular necrosis of the jaws: a growing epidemic. J Oral Maxillofac Surg.

[2] Kawahara M, Kuroshima S, Sawase T (2021). Clinical considerations for medication-related osteonecrosis of the jaw: a comprehensive literature review. Int. J. Implant Dent..

[3] Ruggiero SL, Dodson TB, Aghaloo T, Carlson ER, Ward BB, Kademani D (2022). American Association of oral and maxillofacial surgeons’ position paper on medication-related osteonecrosis of the Jaws-2022 update. J Oral Maxillofac Surg.

[4] Kanwar N, Bakr MM, Meer M, Siddiqi A (2020). Emerging therapies with potential risks of medicine-related osteonecrosis of the jaw: a review of the literature. Br Dent J.

[5] Vanpoecke J, Verstraete L, Smeets M, Ferri J, Nicot R, Politis C (2020). Medication-related osteonecrosis of the jaw (MRONJ) stage III: conservative and conservative surgical approaches versus an aggressive surgical intervention: a systematic review. J Craniomaxillofac Surg.

[6] Kishimoto H, Noguchi K, Takaoka K (2019). Novel insight into the management of bisphosphonate-related osteonecrosis of the jaw (BRONJ). Jpn Dent Sci Rev.

[7] Miller K, Steger GG, Niepel D, Lüftner D (2018). Harnessing the potential of therapeutic agents to safeguard bone health in prostate cancer. Prostate Cancer Prostatic Dis.

[8] Castellano D, Sepulveda JM, García-Escobar I, Rodriguez-Antolín A, Sundlöv A, Cortes-Funes H (2011). The role of RANK-ligand inhibition in cancer: the story of denosumab. Oncologist.

[9] Zhao N, Li QX, Wang YF, Qiao Q, Huang HY, Guo CB (2023). Anti-angiogenic drug aggravates the degree of anti-resorptive drug-based medication-related osteonecrosis of the jaw by impairing the proliferation and migration function of gingival fibroblasts. BMC Oral Health.

[10] McGowan K, McGowan T, Ivanovski S (2018). Risk factors for medication-related osteonecrosis of the jaws: a systematic review. Oral Dis.

[11] Nicolatou-Galitis O, Schiødt M, Mendes RA, Ripamonti C, Hope S, Drudge-Coates L (2019). Medication-related osteonecrosis of the jaw: definition and best practice for prevention, diagnosis, and treatment. Oral Surg Oral Med Oral Pathol Oral Radiol.

[12] Zhang W, Gao L, Ren W, Li S, Zheng J, Li S (2021). The role of the immune response in the development of medication-related osteonecrosis of the jaw. Front Immunol.

[13] Nifosì AF, Zuccarello M, Nifosì L, Hervas Saus V, Nifosì G (2019). Osteonecrosis of the jaw in the era of targeted therapy and immunotherapy in oncology. J Korean Assoc Oral Maxillofac Surg.

[14] Voss PJ, Poxleitner P, Schmelzeisen R, Stricker A, Semper-Hogg W (2017). Update MRONJ and perspectives of its treatment. J Stomatol Oral Maxillofac Surg.

[15] Otto S, Tröltzsch M, Jambrovic V, Panya S, Probst F, Ristow O (2015). Tooth extraction in patients receiving oral or intravenous bisphosphonate administration: a trigger for BRONJ development?. J Craniomaxillofac Surg.

[16] Tresguerres FGF, Torres J, López-Quiles J, Hernández G, Vega JA, Tresguerres IF (2020). The osteocyte: a multifunctional cell within the bone. Ann Anat.

[17] Schwech N, Nilsson J, Gabre P (2023). Incidence and risk factors for medication-related osteonecrosis after tooth extraction in cancer patients-A systematic review. Clin Exp Dent Res.

[18] Moreno-Rabié C, Gaêta-Araujo H, Ferreira-Leite A, Coucke W, Gielen E, Van den Wyngaert T (2024). Local radiographic risk factors for MRONJ in osteoporotic patients undergoing tooth extraction. Oral Dis.

[19] D’Agostino S, Valentini G, Dolci M, Ferrara E (2023). Potential relationship between poor oral hygiene and MRONJ: an observational retrospective Study. Int J Environ Res Publ Health.

[20] Sacco R, Woolley J, Patel G, Calasans-Maia MD, Yates J (2022). Systematic review of medication related osteonecrosis of the jaw (MRONJ) in patients undergoing only antiangiogenic drug therapy: surgery or conservative therapy?. Br J Oral Maxillofac Surg.

[21] Lobekk OK, Dijkstra W, Pedersen T (2021). Surgical vs conservative treatment of medication-related osteonecrosis of the jaw: a complex systematic review and meta-analysis. Oral Surg Oral Med Oral Pathol Oral Radiol.

[22] On SW, Cho SW, Byun SH, Yang BE (2021). Various therapeutic methods for the treatment of Medication-Related Osteonecrosis of the Jaw (MRONJ) and their limitations: a narrative review on new molecular and cellular therapeutic approaches. Antioxidants (Basel).

[23] Khan AA, Morrison A, Hanley DA, Felsenberg D, McCauley LK, O’Ryan F (2015). Diagnosis and management of osteonecrosis of the jaw: a systematic review and international consensus. J Bone Miner Res.

[24] Moore A, Renton T, Taylor T, Popat S, Popat R, Sivardeen Z (2014). Oral surgery: ARONJ masterclass. Br Dent J.

[25] Heck T, Lohana D, Mallela D, Mandil O, Sun L, Saxena P (2024). Hyperbaric oxygen therapy as an adjunct treatment of periodontitis, MRONJ, and ONJ: a systematic literature review. Clin Oral Invest.

[26] Freiberger JJ, Padilla-Burgos R, McGraw T, Suliman HB, Kraft KH, Stolp BW (2012). What is the role of hyperbaric oxygen in the management of bisphosphonate-related osteonecrosis of the jaw: a randomized controlled trial of hyperbaric oxygen as an adjunct to surgery and antibiotics. J Oral Maxillofac Surg.

[27] Kim KM, Kim S, Hwang H, Kim HY, Kim D, Park JH (2024). Effects of daily versus weekly teriparatide for medication-related osteonecrosis of the jaw: a case-control study. Oral Dis.

[28] Jung J, Yoo HY, Kim GT, Lee JW, Lee YA, Kim DY (2017). Short-term teriparatide and recombinant human bone morphogenetic Protein-2 for regenerative approach to medication-related osteonecrosis of the jaw: a preliminary Study. J Bone Miner Res.

[29] Yarom N, Shapiro CL, Peterson DE, Van Poznak CH, Bohlke K, Ruggiero SL (2019). Medication-related osteonecrosis of the Jaw: MASCC/ISOO/ASCO Clinical Practice Guideline. J Clin Oncol.

[30] Bissinger O, Greiser J, Maier E, Ehrmann P, Kakoschke T, Wolff KD (2025). How does medication-related osteonecrosis of the jaw (MRONJ) influence the health-related quality of life after surgery?. BMC Oral Health.

[31] Bragdon B, Moseychuk O, Saldanha S, King D, Julian J, Nohe A (2011). Bone morphogenetic proteins: a critical review. Cell Signal.

[32] Heubel B, Nohe A (2021). The role of BMP signaling in osteoclast regulation. J Dev Biol.

[33] Nakagawa S, Okada R, Kushioka J, Kodama J, Tsukazaki H, Bal Z (2022). Effects of rhBMP-2-loaded hydroxyapatite granules/beta-tricalcium phosphate hydrogel (HA/β-TCP/hydrogel) composite on a rat model of caudal intervertebral fusion. Sci Rep.

[34] Han S, Paeng KW, Park S, Jung UW, Cha JK, Hong J (2021). Programmed BMP-2 release from biphasic calcium phosphates for optimal bone regeneration. Biomaterials.

[35] Taye MB, Ningsih HS, Shih S-J (2024). Exploring the advancements in surface-modified bioactive glass: enhancing antibacterial activity, promoting angiogenesis, and modulating bioactivity. J Nanoparticle Res.

[36] Zhang YS, Khademhosseini A (2017). Advances in engineering hydrogels. Science.

[37] Fan L, Chen S, Yang M, Liu Y, Liu J (2024). Metallic materials for bone repair. Adv Healthcare Mater.

[38] Wood J, Bonjean K, Ruetz S, Bellahcène A, Devy L, Foidart JM (2002). Novel antiangiogenic effects of the bisphosphonate compound zoledronic acid. J Pharmacol Exp Therapeut.

[39] Chen L, Zhou X, He C (2019). Mesoporous silica nanoparticles for tissue-engineering applications. Wiley Interdiscip Rev Nanomed Nanobiotechnol.

[40] Al-Hamed FS, Mahri M, Al-Waeli H, Torres J, Badran Z, Tamimi F (2019). Regenerative effect of platelet concentrates in oral and craniofacial regeneration. Front Cardiovasc Med.

[41] Shan Z, Xie X, Wu X, Zhuang S, Zhang C (2022). Development of degradable magnesium-based metal implants and their function in promoting bone metabolism (A review). J Orthop Transl.

[42] Park JH, Kim JW, Kim SJ (2017). Does the addition of bone morphogenetic protein 2 to platelet-rich fibrin improve healing after treatment for medication-related osteonecrosis of the jaw?. J Oral Maxillofac Surg.

[43] Ferguson SW, Nguyen J (2016). Exosomes as therapeutics: the implications of molecular composition and exosomal heterogeneity. J Contr Release.

[44] Zimta AA, Sigurjonsson OE, Gulei D, Tomuleasa C (2020). The malignant role of exosomes as nanocarriers of rare RNA species. Int J Mol Sci.

[45] Xz Q, Zq S, L L, Hs O (2024). Zoledronic acid accelerates ER stress-mediated inflammation by increasing PDE4B expression in bisphosphonate-related osteonecrosis of the jaw. Appl Biochem Biotechnol.

[46] Lu Y, Huang W, Li M, Zheng A (2023). Exosome-based carrier for RNA delivery: progress and challenges. Pharmaceutics.

[47] Rather MA, Gupta K, Mandal M (2021). Microbial biofilm: formation, architecture, antibiotic resistance, and control strategies. Braz J Microbiol.

[48] Tsoi KM, MacParland SA, Ma XZ, Spetzler VN, Echeverri J, Ouyang B (2016). Mechanism of hard-nanomaterial clearance by the liver. Nat Mater.

[49] Neto AS, Brazete D, Ferreira JMF (2019). Cuttlefish bone-derived biphasic calcium phosphate scaffolds coated with sol-gel derived bioactive glass. Materials (Basel).

[50] Väänänen K (2005). Mechanism of osteoclast mediated bone resorption--rationale for the design of new therapeutics. Adv Drug Deliv Rev.

[51] Shariati K, Bedar M, Huang KX, Moghadam S, Mirzaie S, LaGuardia JS (2025). Biomaterial cues for regulation of osteoclast differentiation and function in bone regeneration. Adv Ther (Weinh).

[52] Seeman E, Martin TJ (2019). Antiresorptive and anabolic agents in the prevention and reversal of bone fragility. Nat Rev Rheumatol.

[53] He L, Sun X, Liu Z, Qiu Y, Niu Y (2020). Pathogenesis and multidisciplinary management of medication-related osteonecrosis of the jaw. Int J Oral Sci.

[54] Wehrhan F, Hyckel P, Guentsch A, Nkenke E, Stockmann P, Schlegel KA (2011). Bisphosphonate-associated osteonecrosis of the jaw is linked to suppressed TGFβ1-signaling and increased Galectin-3 expression: a histological study on biopsies. J Transl Med.

[55] Dong X, He L, Zang X, He Y, An J, Wu B (2021). Adipose-derived stem cells promote bone coupling in bisphosphonate-related osteonecrosis of the jaw by TGF-β1. Front Cell Dev Biol.

[56] Manzano-Moreno FJ, Ramos-Torrecillas J, Melguizo-Rodríguez L, Illescas-Montes R, Ruiz C, García-Martínez O (2018). Bisphosphonate modulation of the gene expression of different markers involved in osteoblast physiology: possible implications in bisphosphonate-related osteonecrosis of the jaw. Int J Med Sci.

[57] Soundia A, Hadaya D, Chau Y, Gkouveris I, Bezouglaia O, Dry S (2021). Local RANKL delivery improves socket healing in bisphosphonate treated rats. Bone.

[58] Kim HJ, Kim HJ, Choi Y, Bae MK, Hwang DS, Shin SH (2019). Zoledronate enhances osteocyte-mediated osteoclast differentiation by IL-6/RANKL axis. Int J Mol Sci.

[59] Maruotti N, Corrado A, Neve A, Cantatore FP (2012). Bisphosphonates: effects on osteoblast. Eur J Clin Pharmacol.

[60] Manzano-Moreno FJ, Ramos-Torrecillas J, De Luna-Bertos E, Ruiz C, García-Martínez O (2015). High doses of bisphosphonates reduce osteoblast-like cell proliferation by arresting the cell cycle and inducing apoptosis. J Craniomaxillofac Surg.

[61] Lotz EM, Lohmann CH, Boyan BD, Schwartz Z (2020). Bisphosphonates inhibit surface-mediated osteogenesis. J Biomed Mater Res A.

[62] Ishtiaq S, Edwards S, Sankaralingam A, Evans BA, Elford C, Frost ML (2015). The effect of nitrogen containing bisphosphonates, zoledronate and alendronate, on the production of pro-angiogenic factors by osteoblastic cells. Cytokine.

[63] Hasmim M, Bieler G, Rüegg C (2007). Zoledronate inhibits endothelial cell adhesion, migration and survival through the suppression of multiple, prenylation-dependent signaling pathways. J Thromb Haemostasis.

[64] Sharma D, Hamlet S, Vaquette C, Petcu EB, Ramamurthy P, Ivanovski S (2021). Local delivery of hydrogel encapsulated vascular endothelial growth factor for the prevention of medication-related osteonecrosis of the jaw. Sci Rep.

[65] Yalcin-Ülker GM, Günbatan M, Duygu G, Soluk-Tekkesin M, Özcakir-Tomruk C (2023). Could local application of hypoxia inducible factor 1-α enhancer deferoxamine be promising for preventing of medication-related osteonecrosis of the jaw?. Biomedicines.

[66] Lee KH, Kim SH, Kim CH, Min BJ, Kim GJ, Lim Y (2019). Identifying genetic variants underlying medication-induced osteonecrosis of the jaw in cancer and osteoporosis: a case control study. J Transl Med.

[67] Li S, Cai X, Guo J, Li X, Li W, Liu Y (2025). Cell communication and relevant signaling pathways in osteogenesis-angiogenesis coupling. Bone Res.

[68] Xie H, Cui Z, Wang L, Xia Z, Hu Y, Xian L (2014). PDGF-BB secreted by preosteoclasts induces angiogenesis during coupling with osteogenesis. Nat Med.

[69] Gao SY, Lin RB, Huang SH, Liang YJ, Li X, Zhang SE (2021). PDGF-BB exhibited therapeutic effects on rat model of bisphosphonate-related osteonecrosis of the jaw by enhancing angiogenesis and osteogenesis. Bone.

[70] Yamashita J, Sawa N, Sawa Y, Miyazono S (2021). Effect of bisphosphonates on healing of tooth extraction wounds in infectious osteomyelitis of the jaw. Bone.

[71] Tsukasaki M, Takayanagi H (2019). Osteoimmunology: evolving concepts in bone-immune interactions in health and disease. Nat Rev Immunol.

[72] Mortara L, Benest AV, Bates DO, Noonan DM (2017). Can the co-dependence of the immune system and angiogenesis facilitate pharmacological targeting of tumours?. Curr Opin Pharmacol.

[73] Yao Y, Cai X, Ren F, Ye Y, Wang F, Zheng C (2021). The macrophage-osteoclast axis in osteoimmunity and osteo-related diseases. Front Immunol.

[74] Zhu W, Xu R, Du J, Fu Y, Li S, Zhang P (2019). Zoledronic acid promotes TLR-4-mediated M1 macrophage polarization in bisphosphonate-related osteonecrosis of the jaw. Faseb j.

[75] Zheng Y, Wang X, He Y, Chen S, He L, Zhang Y (2024). Exosomes from adipose-derived mesenchymal stromal cells prevent medication-related osteonecrosis of the jaw by inhibiting macrophage M1 polarization and pyroptosis. Int J Nanomed.

[76] Yang X, Xu X, Chen J, Wang Q, Wang G, Ai X (2020). Zoledronic acid regulates the synthesis and secretion of IL-1β through histone methylation in macrophages. Cell Death Discov.

[77] Hasiakos S, Gwack Y, Kang M, Nishimura I (2021). Calcium signaling in T cells and chronic inflammatory disorders of the oral cavity. J Dent Res.

[78] Martínez-Corral I, Olmeda D, Diéguez-Hurtado R, Tammela T, Alitalo K, Ortega S (2012). In vivo imaging of lymphatic vessels in development, wound healing, inflammation, and tumor metastasis. Proc Natl Acad Sci USA.

[79] Kim H, Kataru RP, Koh GY (2012). Regulation and implications of inflammatory lymphangiogenesis. Trends Immunol.

[80] Kuroshima S, Yamashita J (2013). Chemotherapeutic and antiresorptive combination therapy suppressed lymphangiogenesis and induced osteonecrosis of the jaw-like lesions in mice. Bone.

[81] Budzinska A, Galganski L, Jarmuszkiewicz W (2023). The bisphosphonates alendronate and zoledronate induce adaptations of aerobic metabolism in permanent human endothelial cells. Sci Rep.

[82] Lu Y, Wang Z, Han W, Li H (2016). Zoledronate induces autophagic cell death in human umbilical vein endothelial cells via Beclin-1 dependent pathway activation. Mol Med Rep.

[83] Qin Z, Xie H, Su P, Song Z, Xu R, Guo S (2024). Targeting endoplasmic reticulum stress-induced lymphatic dysfunction for mitigating bisphosphonate-related osteonecrosis. Clin Transl Med.

[84] Cao Z, Zhao C, Hu L, Peng F, Liu Z, Yang X (2025). Biomaterial-based lymphangiogenesis-enhanced therapeutic strategy for robustly-integrated medication-related osteonecrosis of jaw prevention. ACS Appl Mater Interfaces.

[85] Kalelkar PP, Riddick M, García AJ (2022). Biomaterial-based delivery of antimicrobial therapies for the treatment of bacterial infections. Nat Rev Mater.

[86] Pushalkar S, Li X, Kurago Z, Ramanathapuram LV, Matsumura S, Fleisher KE (2014). Oral microbiota and host innate immune response in bisphosphonate-related osteonecrosis of the jaw. Int J Oral Sci.

[87] Zirk M, Wenzel C, Buller J, Zöller JE, Zinser M, Peters F (2019). Microbial diversity in infections of patients with medication-related osteonecrosis of the jaw. Clin Oral Invest.

[88] Ewald F, Wuesthoff F, Koehnke R, Friedrich RE, Gosau M, Smeets R (2021). Retrospective analysis of bacterial colonization of necrotic bone and antibiotic resistance in 98 patients with medication-related osteonecrosis of the jaw (MRONJ). Clin Oral Invest.

[89] Bassan MMG, Marinho Maciel R, Linhares Ferrazzo K, Cademartori Danesi C (2024). Etiopathogenesis of medication-related osteonecrosis of the jaws: a review. J Mol Med (Berl).

[90] Bouler JM, Pilet P, Gauthier O, Verron E (2017). Biphasic calcium phosphate ceramics for bone reconstruction: a review of biological response. Acta Biomater.

[91] Julien M, Khairoun I, LeGeros RZ, Delplace S, Pilet P, Weiss P (2007). Physico-chemical-mechanical and in vitro biological properties of calcium phosphate cements with doped amorphous calcium phosphates. Biomaterials.

[92] Jeong J, Kim JH, Shim JH, Hwang NS, Heo CY (2019). Bioactive calcium phosphate materials and applications in bone regeneration. Biomater Res.

[93] LeGeros RZ (2008). Calcium phosphate-based osteoinductive materials. Chem Rev.

[94] Li J, Chen YC, Tseng YC, Mozumdar S, Huang L (2010). Biodegradable calcium phosphate nanoparticle with lipid coating for systemic siRNA delivery. J Contr Release.

[95] Liu Y, Hunziker EB, Randall NX, de Groot K, Layrolle P (2003). Proteins incorporated into biomimetically prepared calcium phosphate coatings modulate their mechanical strength and dissolution rate. Biomaterials.

[96] Hou X, Zhang L, Zhou Z, Luo X, Wang T, Zhao X (2022). Calcium phosphate-based biomaterials for bone repair. J Funct Biomater.

[97] Zerbo IR, Bronckers AL, de Lange G, Burger EH (2005). Localisation of osteogenic and osteoclastic cells in porous beta-tricalcium phosphate particles used for human maxillary sinus floor elevation. Biomaterials.

[98] Bohner M, Santoni BLG, Döbelin N (2020). β-tricalcium phosphate for bone substitution: synthesis and properties. Acta Biomater.

[99] Luvizuto ER, Tangl S, Zanoni G, Okamoto T, Sonoda CK, Gruber R (2011). The effect of BMP-2 on the osteoconductive properties of β-tricalcium phosphate in rat calvaria defects. Biomaterials.

[100] Yuan H, Fernandes H, Habibovic P, de Boer J, Barradas AM, de Ruiter A (2010). Osteoinductive ceramics as a synthetic alternative to autologous bone grafting. Proc Natl Acad Sci USA.

[101] Otto S, Hafner S, Mast G, Tischer T, Volkmer E, Schieker M (2010). Bisphosphonate-related osteonecrosis of the jaw: is pH the missing part in the pathogenesis puzzle?. J Oral Maxillofac Surg.

[102] Kameda Y, Aizawa M, Sato T, Honda M (2021). Zoledronic acid-loaded β-TCP inhibits tumor proliferation and osteoclast activation: development of a functional bone substitute for an efficient osteosarcoma treatment. Int J Mol Sci.

[103] Funayama N, Yagyuu T, Imada M, Ueyama Y, Nakagawa Y, Kirita T (2023). Impact of beta-tricalcium phosphate on preventing tooth extraction-triggered bisphosphonate-related osteonecrosis of the jaw in rats. Sci Rep.

[104] Ryan E, Yin S (2022). Compressive strength of β-TCP scaffolds fabricated via lithography-based manufacturing for bone tissue engineering. Ceram Int.

[105] Paulo S, Laranjo M, Paula A, Abrantes AM, Martins J, Marto CM (2020). Calcium phosphate ceramics can prevent bisphosphonate-related osteonecrosis of the jaw. Materials (Basel).

[106] Zhou N, Li Q, Lin X, Hu N, Liao JY, Lin LB (2016). BMP2 induces chondrogenic differentiation, osteogenic differentiation and endochondral ossification in stem cells. Cell Tissue Res.

[107] Mikai A, Ono M, Tosa I, Nguyen HTT, Hara ES, Nosho S (2020). BMP-2/β-TCP local delivery for bone regeneration in MRONJ-like mouse model. Int J Mol Sci.

[108] Bordukalo-Nikšić T, Kufner V, Vukičević S (2022). The role of BMPs in the regulation of osteoclasts resorption and bone remodeling: from experimental models to clinical applications. Front Immunol.

[109] Bhakta G, Lim ZX, Rai B, Lin T, Hui JH, Prestwich GD (2013). The influence of collagen and hyaluronan matrices on the delivery and bioactivity of bone morphogenetic protein-2 and ectopic bone formation. Acta Biomater.

[110] Faßbender M, Minkwitz S, Strobel C, Schmidmaier G, Wildemann B (2014). Stimulation of bone healing by sustained bone morphogenetic protein 2 (BMP-2) delivery. Int J Mol Sci.

[111] Chernysheva M, Ruchko E, Eremeev A (2025). Optimizing rhBMP-2 therapy for bone regeneration: from safety concerns to biomaterial-guided delivery systems. Int J Mol Sci.

[112] Howard MT, Wang S, Berger AG, Martin JR, Jalili-Firoozinezhad S, Padera RF (2022). Sustained release of BMP-2 using self-assembled layer-by-layer film-coated implants enhances bone regeneration over burst release. Biomaterials.

[113] Dang AT, Ono M, Wang Z, Tosa I, Hara ES, Mikai A (2024). Local E-rhBMP-2/β-TCP application rescues osteocyte dendritic integrity and reduces microstructural damage in alveolar bone post-extraction in MRONJ-like mouse model. Int J Mol Sci.

[114] Nguyen HT, Ono M, Oida Y, Hara ES, Komori T, Akiyama K (2019). Bone marrow cells inhibit BMP-2-Induced osteoblast activity in the marrow environment. J Bone Miner Res.

[115] Lombard T, Neirinckx V, Rogister B, Gilon Y, Wislet S (2016). Medication-related osteonecrosis of the jaw: new insights into molecular mechanisms and cellular therapeutic approaches. Stem Cells Int..

[116] El Bialy I, Jiskoot W, Reza Nejadnik M (2017). Formulation, delivery and stability of bone morphogenetic proteins for effective bone regeneration. Pharm Res.

[117] Hoppe A, Güldal NS, Boccaccini AR (2011). A review of the biological response to ionic dissolution products from bioactive glasses and glass-ceramics. Biomaterials.

[118] Zhu Y, Zhang X, Chang G, Deng S, Chan HF (2025). Bioactive glass in tissue regeneration: unveiling recent advances in regenerative strategies and applications. Adv Mater.

[119] Gupta S, Majumdar S, Krishnamurthy S (2021). Bioactive glass: a multifunctional delivery system. J Contr Release.

[120] Skallevold HE, Rokaya D, Khurshid Z, Zafar MS (2019). Bioactive glass applications in dentistry. Int J Mol Sci.

[121] Tabatabaei FS, Torres R, Tayebi L, Tayebi L (2019). Biomedical materials in dentistry. Applications of biomedical engineering in dentistry.

[122] Kaou MH, Furkó M, Balázsi K, Balázsi C (2023). Advanced bioactive glasses: the newest achievements and breakthroughs in the area. Nanomaterials (Basel).

[123] Brauer DS (2015). Bioactive glasses—structure and properties. Angew Chem Int Ed Engl.

[124] Kargozar S, Montazerian M, Hamzehlou S, Kim HW, Baino F (2018). Mesoporous bioactive glasses: promising platforms for antibacterial strategies. Acta Biomater.

[125] Fernandes JS, Gentile P, Pires RA, Reis RL, Hatton PV (2017). Multifunctional bioactive glass and glass-ceramic biomaterials with antibacterial properties for repair and regeneration of bone tissue. Acta Biomater.

[126] Gorustovich AA, Roether JA, Boccaccini AR (2010). Effect of bioactive glasses on angiogenesis: a review of in vitro and in vivo evidences. Tissue Eng Part B Rev.

[127] Yuan H, Niu LN, Jiao K, Pei DD, Pramanik C, Li J (2016). Revival of nitrogen-containing bisphosphonate-induced inhibition of osteoclastogenesis and osteoclast function by water-soluble microfibrous borate glass. Acta Biomater.

[128] Pramanik C, Sood P, Niu LN, Yuan H, Ghoshal S, Henderson W (2016). A mechanistic study of the interaction of water-soluble borate glass with apatite-bound heterocyclic nitrogen-containing bisphosphonates. Acta Biomater.

[129] Ege D, Zheng K, Boccaccini AR (2022). Borate Bioactive Glasses (BBG): bone regeneration, wound healing applications, and future directions. ACS Appl Bio Mater.

[130] Su Z, Li J, Bai X, Tay FR, Zhang M, Liang K (2020). Borate bioactive glass prevents zoledronate-induced osteonecrosis of the jaw by restoring osteogenesis and angiogenesis. Oral Dis.

[131] Pellicano AA, Benites BM, Paschoa AFN, Oliveira LC, Campos ACP, Martins DO (2024). Mitigating jaw osteonecrosis: bioactive glass and pericardial membrane combination in a rat model. Front Oncol.

[132] Franz S, Rammelt S, Scharnweber D, Simon JC (2011). Immune responses to implants - a review of the implications for the design of immunomodulatory biomaterials. Biomaterials.

[133] Azizi L, Turkki P, Huynh N, Massera JM, Hytönen VP (2021). Surface modification of bioactive glass promotes cell attachment and spreading. ACS Omega.

[134] Liu J, Yang L, Liu K, Gao F (2023). Hydrogel scaffolds in bone regeneration: their promising roles in angiogenesis. Front Pharmacol.

[135] Lin H, Yin C, Mo A, Hong G (2021). Applications of hydrogel with special physical properties in bone and cartilage regeneration. Materials (Basel).

[136] Yuan Z, Wei P, Huang Y, Zhang W, Chen F, Zhang X (2019). Injectable PLGA microspheres with tunable magnesium ion release for promoting bone regeneration. Acta Biomater.

[137] Qin S, Yuan H, Shan Z, Wang J, Pan W (2026). Rational design of mechanically optimized hydrogels for bone tissue engineering: a review. Gels.

[138] Bose S, Roy M, Bandyopadhyay A (2012). Recent advances in bone tissue engineering scaffolds. Trends Biotechnol.

[139] Wu G, Feng C, Quan J, Wang Z, Wei W, Zang S (2018). In situ controlled release of stromal cell-derived factor-1α and antimiR-138 for on-demand cranial bone regeneration. Carbohydr Polym.

[140] Zara JN, Siu RK, Zhang X, Shen J, Ngo R, Lee M (2011). High doses of bone morphogenetic protein 2 induce structurally abnormal bone and inflammation in vivo. Tissue Eng Part A.

[141] Shirakura T, Smith C, Hopkins TJJ, Koo Lee YE, Lazaridis F, Argyrakis P (2017). Matrix density engineering of hydrogel nanoparticles with simulation-guided synthesis for tuning drug release and cellular uptake. ACS Omega.

[142] Brierly GI, Ren J, Baldwin J, Saifzadeh S, Theodoropoulos C, Tsurkan MV (2019). Investigation of sustained BMP delivery in the prevention of Medication-Related Osteonecrosis of the Jaw (MRONJ) in a rat model. Macromol Biosci.

[143] Thavornyutikarn B, Sungkhaphan P, Kaewkong P, Pornsuwan S, Risangud N, Singhatanadgit W (2023). Biodegradable dual-function nanocomposite hydrogels for prevention of bisphosphonate-related osteonecrosis of the jaw. ACS Appl Bio Mater.

[144] Li J, Mooney DJ (2016). Designing hydrogels for controlled drug delivery. Nat Rev Mater.

[145] Bai L, Tao G, Feng M, Xie Y, Cai S, Peng S (2023). Hydrogel drug delivery systems for bone regeneration. Pharmaceutics.

[146] Yang X, Li S, Ren Y, Qiang L, Liu Y, Wang J (2022). 3D printed hydrogel for articular cartilage regeneration. Compos Part B Eng.

[147] Li Y, Liu J, Wei J, Yuan L, Hu J, Dai S (2025). Porous hydrogels prepared by two-step gelation method for bone regeneration. J Funct Biomater.

[148] Chen Q, Thouas GA (2015). Metallic implant biomaterials. Mater Sci Eng R Rep.

[149] Zhou Y, Zhang A, Wu J, Guo S, Sun Q (2024). Application and perspectives: magnesium materials in bone regeneration. ACS Biomater Sci Eng.

[150] Chen Y, Zhang S, Bai J, Yang Y, Wang Y, Zhou Y (2024). Magnesium-based biomaterials for coordinated tissue repair: a comprehensive overview of design strategies, advantages, and challenges. J Magnesium Alloys.

[151] Wang JL, Xu JK, Hopkins C, Chow DH, Qin L (2020). Biodegradable magnesium-based implants in Orthopedics-A general review and perspectives. Adv Sci (Weinh).

[152] Esmaily M, Svensson JE, Fajardo S, Birbilis N, Frankel GS, Virtanen S (2017). Fundamentals and advances in magnesium alloy corrosion. Prog Mater Sci.

[153] Wu J, Cheng X, Wu J, Chen J, Pei X (2024). The development of magnesium-based biomaterials in bone tissue engineering: a review. J Biomed Mater Res B Appl Biomater.

[154] Chen L, Yan Z, Qiu T, Zhu J, Liu G, Han J (2023). Long-term temporospatial complementary relationship between degradation and bone regeneration of Mg-Al alloy. ACS Appl Bio Mater.

[155] Zhou W, Yan J, Li Y, Wang L, Jing L, Li M (2021). Based on the synergistic effect of Mg(2+) and antibacterial peptides to improve the corrosion resistance, antibacterial ability and osteogenic activity of magnesium-based degradable metals. Biomater Sci.

[156] Witte F (2010). The history of biodegradable magnesium implants: a review. Acta Biomater.

[157] Yan ZY, Zhu JH, Liu GQ, Liu ZC, Guo CB, Cui NH (2022). Feasibility and efficacy of a degradable magnesium-alloy GBR membrane for bone augmentation in a distal bone-defect model in beagle dogs. Bioinorg Chem Appl.

[158] Zhang X, Zu H, Zhao D, Yang K, Tian S, Yu X (2017). Ion channel functional protein kinase TRPM7 regulates Mg ions to promote the osteoinduction of human osteoblast via PI3K pathway: in vitro simulation of the bone-repairing effect of Mg-based alloy implant. Acta Biomater.

[159] Zhu WY, Guo J, Yang WF, Tao ZY, Lan X, Wang L (2022). Biodegradable magnesium implant enhances angiogenesis and alleviates medication-related osteonecrosis of the jaw in rats. J Orthop Transl.

[160] Allo BA, Costa DO, Dixon SJ, Mequanint K, Rizkalla AS (2012). Bioactive and biodegradable nanocomposites and hybrid biomaterials for bone regeneration. J Funct Biomater.

[161] Das S, Vishakha K, Banerjee S, Nag D, Ganguli A (2022). Tetracycline-loaded magnesium oxide nanoparticles with a potential bactericidal action against multidrug-resistant bacteria: in vitro and in vivo evidence. Colloids Surf B Biointerfaces.

[162] Kim SM, Patel M, Patel R (2021). PLGA core-shell nano/microparticle delivery system for biomedical application. Polymers (Basel).

[163] del Mercato LL, Ferraro MM, Baldassarre F, Mancarella S, Greco V, Rinaldi R (2014). Biological applications of LbL multilayer capsules: from drug delivery to sensing. Adv Colloid Interface Sci.

[164] Guo J, Yao H, Chang L, Zhu W, Zhang Y, Li X (2025). Magnesium nanocomposite hydrogel reverses the pathologies to enhance mandible regeneration. Adv Mater.

[165] Ghosh S, Webster TJ (2021). Mesoporous silica based nanostructures for bone tissue regeneration. Front Mater.

[166] Deshpande S, Sharma S, Koul V, Singh N (2017). Core-shell nanoparticles as an efficient, sustained, and triggered drug-delivery system. ACS Omega.

[167] Bovari-Biri J, Miskei JA, Kover Z, Steinerbrunner-Nagy A, Kardos K, Maroti P (2025). Advancements in bone replacement techniques-potential uses after maxillary and mandibular resections due to Medication-Related Osteonecrosis of the Jaw (MRONJ). Cells.

[168] De Koker S, Hoogenboom R, De Geest BG (2012). Polymeric multilayer capsules for drug delivery. Chem Soc Rev.

[169] Mijiritsky E, Assaf HD, Peleg O, Shacham M, Cerroni L, Mangani L (2021). Use of PRP, PRF and CGF in periodontal regeneration and facial Rejuvenation-A narrative review. Biology (Basel)..

[170] Dohan Ehrenfest DM, Rasmusson L, Albrektsson T (2009). Classification of platelet concentrates: from pure platelet-rich plasma (P-PRP) to leucocyte- and platelet-rich fibrin (L-PRF). Trends Biotechnol.

[171] Yi K, Li Q, Lian X, Wang Y, Tang Z (2022). Utilizing 3D bioprinted platelet-rich fibrin-based materials to promote the regeneration of oral soft tissue. Regen Biomater.

[172] Song P, He D, Ren S, Fan L, Sun J (2024). Platelet-rich fibrin in dentistry. J Appl Biomater Funct Mater.

[173] Qiu M, He Y, Zhang H, Zheng Y, Shi X, Yang J (2024). Platelet-rich plasma (PRP) based on simple and efficient integrated preparation precises quantitatively for skin wound repair. Int J Mol Sci.

[174] Tao L, Gao Y, Li Y, Yang L, Yao J, Huang H (2024). The preventive effect of photocrosslinked Hep/GelMA hydrogel loaded with PRF on MRONJ. BMC Oral Health.

[175] Asaka T, Ohga N, Yamazaki Y, Sato J, Satoh C, Kitagawa Y (2017). Platelet-rich fibrin may reduce the risk of delayed recovery in tooth-extracted patients undergoing oral bisphosphonate therapy: a trial study. Clin Oral Invest.

[176] Cortese A, Casarella A, Howard CM, Claudio PP (2021). Epi-mucosa fixation and autologous platelet-rich fibrin treatment in medication-related osteonecrosis of the jaw. Dent J (Basel).

[177] Fernando de Almeida Barros Mourão C, Calasans-Maia MD, Del Fabbro M, Le Drapper Vieira F, Coutinho de Mello Machado R, Capella R (2020). The use of Platelet-rich Fibrin in the management of medication-related osteonecrosis of the jaw: a case series. J Stomatol Oral Maxillofac Surg.

[178] Alrmali A, Saleh MHA, Kurdi SMS, Sabri H, Meghil MM, Wang HL (2023). Prevention and management of drug-induced osteonecrosis of the jaws using platelet-rich fibrin: a clinical feasibility study. Clin Exp Dent Res.

[179] Maracineanu R, Tudor A, Hum I, Urtila F, Streian F, Talpos-Niculescu S (2025). Platelet-rich fibrin in MRONJ management: a prospective comparative study on its effectiveness in prevention and treatment. Medicina (Kaunas).

[180] Reis CHB, Buchaim DV, Ortiz AC, Fideles SOM, Dias JA, Miglino MA (2022). Application of fibrin associated with photobiomodulation as a promising strategy to improve regeneration in tissue engineering: a systematic review. Polymers (Basel).

[181] Bouland CL, Javadian R, Gilis S, Yanni A, Le Clercq M, Mestrallet P (2024). Treatment of medication-related osteonecrosis of the jaw with cell therapy. Front Cell Dev Biol.

[182] Zang X, He L, Zhao L, He Y, Xiao E, Zhang Y (2019). Adipose-derived stem cells prevent the onset of bisphosphonate-related osteonecrosis of the jaw through transforming growth factor β-1-mediated gingival wound healing. Stem Cell Res Ther.

[183] Liao L, Shi B, Chang H, Su X, Zhang L, Bi C (2017). Heparin improves BMSC cell therapy: anticoagulant treatment by heparin improves the safety and therapeutic effect of bone marrow-derived mesenchymal stem cell cytotherapy. Theranostics.

[184] Zheng Y, Dong X, Wang X, Wang J, Chen S, He Y (2023). Exosomes derived from adipose tissue-derived mesenchymal stromal cells prevent medication-related osteonecrosis of the jaw through IL-1RA. Int J Mol Sci.

[185] Herrmann IK, Wood MJA, Fuhrmann G (2021). Extracellular vesicles as a next-generation drug delivery platform. Nat Nanotechnol.

[186] Cheng L, Hill AF (2022). Therapeutically harnessing extracellular vesicles. Nat Rev Drug Discov.

[187] Huang J, Wang L, Tian W (2021). Small extracellular vesicles derived from adipose tissue prevent bisphosphonate-related osteonecrosis of the jaw by promoting angiogenesis. Int J Nanomed.

[188] Zhang Y, Yu M, Dai M, Chen C, Tang Q, Jing W (2017). miR-450a-5p within rat adipose tissue exosome-like vesicles promotes adipogenic differentiation by targeting WISP2. J Cell Sci.

[189] Watanabe J, Sakai K, Urata Y, Toyama N, Nakamichi E, Hibi H (2020). Extracellular vesicles of stem cells to prevent BRONJ. J Dent Res.

[190] Zhang Y, Liu Q, Zhang X, Huang H, Tang S, Chai Y (2022). Recent advances in exosome-mediated nucleic acid delivery for cancer therapy. J Nanobiotechnol.

[191] Ela S, Mäger I, Breakefield XO, Wood MJ (2013). Extracellular vesicles: biology and emerging therapeutic opportunities. Nat Rev Drug Discov.

[192] Xu J, Wang Y, Hsu CY, Gao Y, Meyers CA, Chang L (2019). Human perivascular stem cell-derived extracellular vesicles mediate bone repair. eLife.

[193] Law YY, Lee WF, Hsu CJ, Lin YY, Tsai CH, Huang CC (2021). miR-let-7c-5p and miR-149-5p inhibit proinflammatory cytokine production in osteoarthritis and rheumatoid arthritis synovial fibroblasts. Aging (Albany NY).

[194] Musolino C, Oteri G, Allegra A, Mania M, D’Ascola A, Avenoso A (2018). Altered microRNA expression profile in the peripheral lymphoid compartment of multiple myeloma patients with bisphosphonate-induced osteonecrosis of the jaw. Ann Hematol.

[195] Shen X, Zhu W, Zhang P, Fu Y, Cheng J, Liu L (2022). Macrophage miR-149-5p induction is a key driver and therapeutic target for BRONJ. JCI Insight.

[196] Ahn SH, Ryu SW, Choi H, You S, Park J, Choi C (2022). Manufacturing therapeutic exosomes: from bench to industry. Mol Cells.

[197] Witwer KW, Goberdhan DC, O’Driscoll L, Théry C, Welsh JA, Blenkiron C (2021). Updating MISEV: evolving the minimal requirements for studies of extracellular vesicles. J Extracell Vesicles.

[198] Pegtel DM, Gould SJ (2019). Exosomes. Annu Rev Biochem.

[199] Gimona M, Pachler K, Laner-Plamberger S, Schallmoser K, Rohde E (2017). Manufacturing of human extracellular vesicle-based therapeutics for clinical use. Int J Mol Sci.

[200] Qu Q, Fu B, Long Y, Liu ZY, Tian XH (2023). Current strategies for promoting the large-scale production of exosomes. Curr Neuropharmacol.

[201] Haghighitalab A, Dominici M, Matin MM, Shekari F, Ebrahimi Warkiani M, Lim R (2023). Extracellular vesicles and their cells of origin: open issues in autoimmune diseases. Front Immunol.

[202] Tetradis S, Allen MR, Ruggiero SL (2023). Pathophysiology of medication-related osteonecrosis of the Jaw-A minireview. JBMR Plus.

[203] Lin Y, Li Q, Wang L, Guo Q, Liu S, Zhu S (2022). Advances in regenerative medicine applications of tetrahedral framework nucleic acid-based nanomaterials: an expert consensus recommendation. Int J Oral Sci.

[204] Cui W, Chen X, Zhu J, Zhang M, Xiao D, Qin X (2020). Preventive effect of tetrahedral framework nucleic acids on bisphosphonate-related osteonecrosis of the jaw. Nanoscale.

[205] Zhang T, Tian T, Lin Y (2022). Functionalizing framework nucleic-acid-based nanostructures for biomedical application. Adv Mater.

[206] Zhao D, Xiao D, Liu M, Li J, Peng S, He Q (2022). Tetrahedral framework nucleic acid carrying angiogenic peptide prevents bisphosphonate-related osteonecrosis of the jaw by promoting angiogenesis. Int J Oral Sci.

[207] Zelepukin IV, Shevchenko KG, Deyev SM (2024). Rediscovery of mononuclear phagocyte system blockade for nanoparticle drug delivery. Nat Commun.

[208] Desai N, Rana D, Patel M, Bajwa N, Prasad R, Vora LK (2025). Nanoparticle therapeutics in clinical perspective: classification, marketed products, and regulatory landscape. Small.

[209] Dobrovolskaia MA, Aggarwal P, Hall JB, McNeil SE (2008). Preclinical studies to understand nanoparticle interaction with the immune system and its potential effects on nanoparticle biodistribution. Mol Pharm.

[210] Salamanca E, Pan YH, Sun YS, Hsueh HW, Dorj O, Yao WL (2022). Magnesium modified β-Tricalcium phosphate induces cell osteogenic differentiation in vitro and bone regeneration in vivo. Int J Mol Sci.

[211] Hadad H, Kawamata de Jesus L, Piquera Santos AF, Rinaldi MH, de Souza Rodrigues LG, Paolo Poli P (2022). Beta tricalcium phosphate, either alone or in combination with antimicrobial photodynamic therapy or doxycycline, prevents medication-related osteonecrosis of the jaw. Sci Rep.

[212] Mao Y, Xu Z, He Z, Wang J, Zhu Z (2023). Wet-adhesive materials of oral and maxillofacial region: from design to application. Chin Chem Lett.

[213] Yan R, Jiang R, Hu L, Deng Y, Wen J, Jiang X (2022). Establishment and assessment of rodent models of medication-related osteonecrosis of the jaw (MRONJ). Int J Oral Sci.

[214] Hanada D, Mochizuki M, Nakahara T, Tanaka A (2025). Novel standardized method for inducing medication-related osteonecrosis of the jaw in rats and precise quantitative assessment of pathological outcomes. Odontology.

[215] Valic MS, Zheng G (2019). Research tools for extrapolating the disposition and pharmacokinetics of nanomaterials from preclinical animals to humans. Theranostics.

[216] Mosaddad SA, Hussain A, Tebyaniyan H (2024). Exploring the use of animal models in craniofacial regenerative medicine: a narrative review. Tissue Eng Part B Rev.

[217] Titsinides S, Karatzas T, Perrea D, Eleftheriadis E, Podaropoulos L, Kalyvas D (2020). Osseous healing in surgically prepared bone defects using different grafting materials: an experimental study in pigs. Dent J (Basel)..

[218] Messer JG, Mendieta Calle JL, Jiron JM, Castillo EJ, Van Poznak C, Bhattacharyya N (2018). Zoledronic acid increases the prevalence of medication-related osteonecrosis of the jaw in a dose dependent manner in rice rats (Oryzomys palustris) with localized periodontitis. Bone.

[219] Gillman CE, Jayasuriya AC (2021). FDA-approved bone grafts and bone graft substitute devices in bone regeneration. Mater Sci Eng C Mater Biol Appl.

[220] Del Toro Runzer C, Balmayor ER, van Griensven M (2026). Biologics for bone regeneration: advances in cell, protein, gene, and mRNA therapies. Bone Res.

